# TREX2 enables efficient genome disruption mediated by paired CRISPR-Cas9 nickases that generate 3′-overhanging ends

**DOI:** 10.1016/j.omtn.2023.102072

**Published:** 2023-11-02

**Authors:** Yue Wang, Yi-Li Feng, Qian Liu, Jing-Jing Xiao, Si-Cheng Liu, Zhi-Cheng Huang, An-Yong Xie

**Affiliations:** 1 Key Laboratory of Laparoscopic Technology of Zhejiang Province, Department of General Surgery, Sir Run-Run Shaw Hospital, Zhejiang University School of Medicine, Hangzhou 310016, P.R. China; 2 Hangzhou Qiantang Hospital, Hangzhou, Zhejiang 310018, P.R. China; 3 Institute of Translational Medicine, Zhejiang University School of Medicine and Zhejiang University Cancer Center, Hangzhou, Zhejiang 310029, P.R. China

**Keywords:** MT: RNA/DNA Editing, paired SpCas9 nickases, TREX2, genome disruption, off-target effect, SpCas9n-TREX2 fusion

## Abstract

Paired *Sp*Cas9 nickases (*Sp*Cas9n) are an effective strategy to reduce off-target effect in genome editing. However, this approach is not efficient with 3′-overhanging ends, limiting its applications. In order to expand the utility of paired *Sp*Cas9n in genome editing, we tested the effect of the TREX2 3′-5′ exonuclease on repair of 3′-overhanging ends. We found ectopic overexpression of *Trex2* stimulates the efficiency of paired *Sp*Cas9n in genome disruption with 3′-overhanging ends up to 400-fold with little stimulation of off-target editing. TREX2 overexpressed preferentially deletes entire 3′ overhangs but has no significant effect on 5′ overhangs. *Trex2* overexpression also stimulates genome disruption by paired *Sp*Cas9n that potentially generate short 3′-overhanging ends at overlapping *Sp*Cas9n target sites, suggesting sequential nicking of overlapping target sites by *Sp*Cas9n. This approach is further simplified with improved efficiency and safety by fusion of TREX2 and particularly its DNA-binding-deficient mutant to *Sp*Cas9n. Junction analysis at overlapping targets revealed the different extent of end resection of 3′ single-stranded DNA (ssDNA) by free TREX2 and TREX2 fused to *Sp*Cas9n. *Sp*Cas9n-TREX2 fusion is more convenient and safer than overexpression of free TREX2 to process 3′-overhanging ends for efficient genome disruption by paired *Sp*Cas9n, allowing practical use of this TREX2-based strategy in genome editing.

## Graphical abstract

**Figure:**
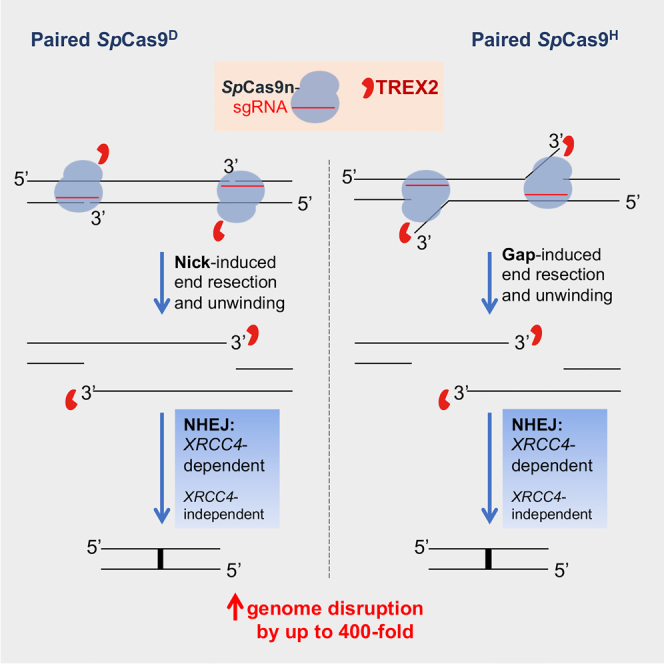

## Teaser

Paired Cas9 nickases (Cas9ns) that generate 3′-overhanging ends are inefficient in genome editing. By engineering TREX2 to degrade 3′ overhangs generated by paired Cas9ns, the corresponding author and colleagues develop a method to stimulate paired Cas9n-induced genome disruption up to 400-fold, thus expanding the utility of paired Cas9n in genome editing.

## Introduction

*Streptococcus pyogenes* Cas9 (*Sp*Cas9), guided by single guide RNA (sgRNA), is presently one of the widely employed programmable nucleases in targeted CRISPR genome editing with broad applications.^1^^,^^2^ *Sp*Cas9 relies on its two nuclease domains (RuvC and HNH) to cleave opposite strands of the DNA target and create a blunt-ended DNA double-strand break (DSB). Subsequent repair of this site-specific DSB is mediated by two major DSB repair pathways, homology-directed repair (HDR) and non-homologous end joining (NHEJ).^3^ Using homologous sequences as a template, HDR is the preferred pathway for accurate substitutions and insertions in CRISPR-Cas9 genome editing, whereas NHEJ is mostly used for gene disruption. NHEJ can be further divided into two sub-pathways, the primary classical NHEJ (c-NHEJ), which requires the core factors such as DNA-PKcs, Ku70/Ku80, XRCC4, and DNA ligase 4, and alternative end joining (a-EJ), which operates without involvement of either c-NHEJ core factor.^4^^,^^5^ Although a-EJ is more error prone than c-NHEJ, both can induce insertions and deletions (indels) in the NHEJ products defined as mutagenic NHEJ (m-NHEJ) products.^5^^,^^6^^,^^7^ Some of these on-target m-NHEJ products are products of interest. Previous studies have demonstrated that c-NHEJ is intrinsically accurate in repair of *Sp*Cas9-induced DSBs, the ends of which are readily ligatable.^8^^,^^9^^,^^10^ However, it is now well recognized that repeated cleavage of accurate repair products by *Sp*Cas9 leads to highly efficient gene disruption.^7^^,^^8^^,^^11^^,^^12^^,^^13^^,^^14^

The *Sp*Cas9-sgRNA complex may also bind to off-target sites and induce DSBs outside the intended target sites, thus generating off-target mutations.^15^^,^^16^ This off-target effect limits the utility of *Sp*Cas9 in genome-editing applications and particularly raises a serious safety concern for clinical applications. Many efforts have been made to reduce such off-target effects while retaining the on-target editing efficiency.^15^^,^^16^^,^^17^^,^^18^^,^^19^^,^^20^ Among the approaches developed, the *Sp*Cas9 nickase (*Sp*Cas9n) mutant D10A (Cas9^D^) or H840A (Cas9^H^) has been used with a pair of sgRNAs for double nicking of opposite strands at a target, creating a DSB with 5′- or 3′-overhanging ends for genome modifications with reduced probability at off-target sites.^20^^,^^21^^,^^22^^,^^23^ Compared with *Sp*Cas9, paired *Sp*Cas9n improved the specificity of targeted gene disruption by up to 1,500-fold. In one genome-wide screening study, the paired Cas9n method even reduced 33 detectable off-target hotspots for a target by *Sp*Cas9 to zero in the genome of human 293T cells.^24^ However, in the paired *Sp*Cas9n method, *Sp*Cas9 D10A (*Sp*Cas9^D^) is often much more effective than *Sp*Cas9 H840A (*Sp*Cas9^H^), likely due to their different cleaving activities.^20^ More importantly, this method is only efficient with 5′-overhanging ends in gene disruption, not with 3′-overhanging ends, thus limiting its application. While paired *Sp*Cas9^D^ are frequently used in genome editing,^3^^,^^16^^,^^20^^,^^25^^,^^26^^,^^27^^,^^28^ enabling the workability with 3′-overhanging ends would broaden the utility of the paired *Sp*Cas9n method.

Although repeated cleavage of a target by *Sp*Cas9 helps accumulate insertions and deletions (indels) in genome editing, intrinsically accurate repair by c-NHEJ remains a barrier to overcome for more efficient targeted gene disruption and HDR-mediated genome editing. Coupling with expression of exonucleases such as TREX2, ExoI, and ExoIII has been exploited to enhance targeted mutagenesis by *Sp*Cas9.^12^^,^^14^^,^^29^^,^^30^^,^^31^^,^^32^ In fact, this strategy had been tested earlier for ZFN and TALEN.^33^^,^^34^ ZFN, TALEN, and *Sp*Cas9 as well as the homing endonuclease I-SceI generate ends with different overhang polarities, such as 4-nt 3′ overhangs for I-SceI, blunt ends for *Sp*Cas9, 4-5-nt 5′ overhangs for ZFN, and heterogeneous short overhangs for TALEN. Because TREX2 is a non-processive 3′-5′ exonuclease that removes 3′ ssDNA or mismatched sequences,^35^^,^^36^ it is surprising that TREX2 increases the frequency of targeted indels induced by these endonucleases that generate blunt ends or 5′ overhangs.^31^^,^^32^^,^^34^^,^^37^^,^^38^ Understanding the end processing of 5′ DSBs and DSBs with blunt ends as well as 3′ DSBs by TREX2 may help tailor CRISPR-Cas9 for better efficiency and specificity. While co-expression of exogenous TREX2 with *Sp*Cas9 was implemented to increase targeted mutagenesis quickly after the induction of CRISPR-Cas9 genome editing,^14^^,^^31^ fusion of TREX2 to *Sp*Cas9 was recently used to promote targeted gene disruption while suppressing translocations.^12^^,^^39^ The safety concern of using TREX2 in genome editing was further alleviated by the observation that TREX2 co-expression or *Sp*Cas9-TREX2 fusion proteins cause no overt toxicity to cells.^12^^,^^34^^,^^39^^,^^40^

In the paired *Sp*Cas9n-sgRNA method, paired nicks on opposite strands are expected to generate clean ends with complementary 5′ overhangs or 3′ overhangs; however, it is still poorly understood how the opposite strands are separated from nicks to generate a DSB with overhanging ends.^3^^,^^20^^,^^25^ It is possible that this strand separation may be mediated by DNA unwinding or end resection involving helicases and nucleases. An attempt has been made with the help of TREX2 to interrogate the strand separation after induction of paired nicks on opposite strands, yielding a model to explain why NHEJ repair of staggered ends with complementary 5′ overhangs induces a much higher level of targeted mutagenesis than 3′ overhangs.^25^ In addition, as paired *Sp*Cas9^H^s have recently been employed to induce nicks on opposite strands in the development of prime editor 3 (PE3) and paired prime editors,^17^^,^^41^^,^^42^^,^^43^^,^^44^^,^^45^ better understanding of the DNA damage response and repair induced by these paired nicks may help further improve prime editing for small edits and long indels.

Here, increasing the editing efficiency with 3′-overhanging ends is our priority to overcome the limitation of the paired *Sp*Cas9n method and broaden its utility. Given the 3′-5′ exonuclease activity of TREX2 and the stimulatory effect of *Trex2* overexpression on m-NHEJ of I-SceI-induced DSBs with 4-nt 3′ overhangs, we tested the effect of *Trex2* co-expression and TREX2 fusion on repair of 3′-overhanging ends generated by paired *Sp*Cas9n on non-overlapping and overlapping targets. We found that both *Trex2* co-expression and TREX2 fusion stimulate the efficiency of paired *Sp*Cas9n in genome disruption with 3′-overhanging ends. In particular, the *Sp*Cas9n-TREX2 fusion serves as a simplified version of the paired Cas9n-sgRNA approach, with more convenient use and less safety concern. Moreover, analysis of off-target effect revealed that *Trex2* overexpression causes little stimulation in off-target editing by paired *Sp*Cas9n. Taken together, this study provides a strategy to enable efficient genome disruption by paired *Sp*Cas9n generating 3′-overhanging ends, broadening the utility of paired *Sp*Cas9n in genome editing.

## Results

### *Trex2* overexpression stimulates m-NHEJ of 3′-overhanging ends generated by paired *Sp*Cas9n-sgRNAs

**Figure 1:**
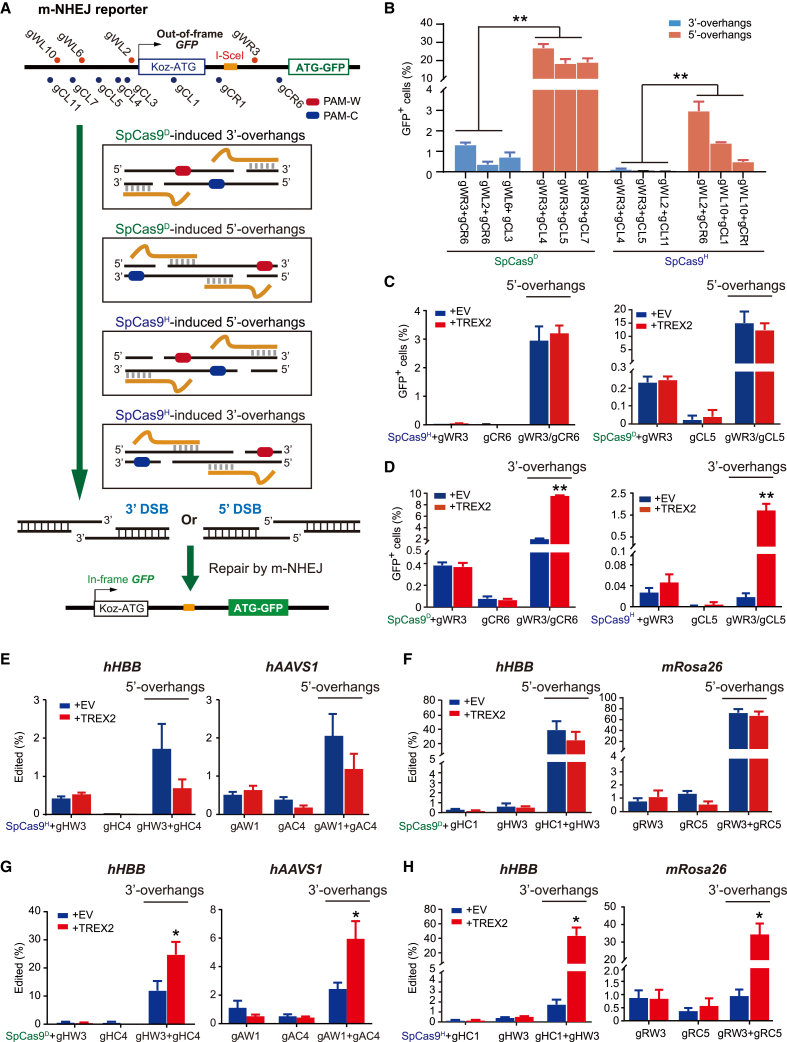
*Trex2* overexpression stimulates m-NHEJ of 3′-overhanging ends induced by paired *Sp*Cas9n (A) Schematic of the m-NHEJ reporter for repair of DSBs with 5′ or 3′ overhangs generated by paired *Sp*Cas9^D^ or paired *Sp*Cas9^H^ that nick opposite strands. Translation from the Koz-ATG in the m-NHEJ reporter normally generates a frameshift *GFP* gene. Site-specific DSBs with 5′ or 3′ overhangs induced by paired *Sp*Cas9n are repaired by m-NHEJ and this repair could correct the *GFP* reading frame and generate GFP^+^ cells. (B) The efficiency of paired *Sp*Cas9n-induced m-NHEJ represented by induced GFP^+^ cells. Paired sgRNAs with *Sp*Cas9^D^ and *Sp*Cas9^H^ generated DSBs with 5′ or 3′ overhangs as indicated. (C and D) Effect of *Trex2* overexpression on m-NHEJ induced by single or paired *Sp*Cas9n in the m-NHEJ reporter. Paired sgRNAs with *Sp*Cas9^D^ and *Sp*Cas9^H^ generated DSBs with 5′ overhangs (C) or 3′ overhangs (D) as indicated. (E–H) Effect of *Trex2* overexpression on targeted gene disruption induced by single or paired *Sp*Cas9n. DSBs with 5′ overhangs were generated by *Sp*Cas9^H^ with gHW3/gHC4 at the *hHBB* locus and gAW1/gAC4 at the *hAAVS1* locus (E) and *Sp*Cas9^D^ with gHC1/gHW3 at the *hHBB* locus and gRW3/gRC5 at the *mRosa26* locus (F), and DSBs with 3′ overhangs generated by *Sp*Cas9^D^ with gHW3/gHC4 at the *hHBB* locus and gAW1/gAC4 at the *hAAVS1* locus (G) and *Sp*Cas9^H^ with gHC1/gHW3 at the *hHBB* locus and gRW3/gRC5 at the *mRosa26* locus (H), as indicated. Columns indicate the mean ± SEM from three independent experiments. Statistics were performed by one-way ANOVA in (B) and by two-tailed Student’s t test in (D), (G), and (H). ∗p < 0.05, ∗∗p < 0.01.

Previously, we established mouse embryonic stem (ES) cells containing a single-copy m-NHEJ reporter, in which only m-NHEJ of a site-specific DSB can lead to GFP^+^ cells.^7^ Using the same reporter cells, we first used I-SceI, *Sp*Cas9, *Sa*Cas9, or *Lb*Cas12a to induce site-specific DSBs at different sites respectively with 3′-overhanging ends, blunt ends, or 5′-overhanging ends in the reporter and analyzed the effect of TREX2 on NHEJ of these different ends (Figure S1A). *Trex2* overexpression stimulated m-NHEJ induced by I-SceI and *Sp*Cas9 (Figures S1B–S1D; Table S1), consistent with previous studies.^31^^,^^32^^,^^34^^,^^37^ Similarly, *Trex2* overexpression stimulated m-NHEJ induced by *Sa*Cas9. The stimulation was more significant for I-SceI that generates 3′-overhanging ends than for *Sp*Cas9 and *Sa*Cas9 that generate blunt ends (Figures S1B–S1D; Table S1). In contrast, *Trex2* overexpression had no effect on *Lb*Cas12a-induced m-NHEJ (Figures S1B–S1D; Table S1), possibly due to 5′ overhangs generated by *Lb*Cas12a. We then used paired *Sp*Cas9n-sgRNAs to induce nicks on opposite strands and generate site-specific DSBs with complementary 5′- or 3′-overhanging ends in m-NHEJ reporter mouse ES cells (Figures 1A and S2A). Similar to a previous study,^20^ m-NHEJ of 3′-overhanging ends is inefficient regardless of *Sp*Cas9D10A (*Sp*Cas9^D^) or *Sp*Cas9H840A (*Sp*Cas9^H^), whereas m-NHEJ of 5′-overhanging ends is over 15% in efficiency for *Sp*Cas9^D^ and 0.4%–3.5% for *Sp*Cas9^H^ (Figure 1B; Table S1). Also as expected, *Trex2* overexpressed did not stimulate m-NHEJ induced by paired *Sp*Cas9^H^-gWR3/gCR6 that generated DSB ends with 14-nt 5′ overhangs, and by paired *Sp*Cas9^D^-gWR3/gCL5 that generated DSB ends with 67-nt 5′ overhangs (Figure 1C; Table S1). However, *Trex2* overexpression increased the m-NHEJ frequency for paired *Sp*Cas9^D^-gWR3/gCR6 with 14-nt 3′ overhangs by up to 6.7-fold and for paired *Sp*Cas9^H^-gWR3/gCL5 with 67-nt 3′ overhangs by up to 86.1-fold (Figure 1D; Table S1). As a comparison, the level of GFP^+^ cells induced by single nicks was minimal and not affected by TREX2 (Figures 1C and 1D; Table S1).

We also used targeted amplicon deep sequencing to directly measure the frequency of targeted insertions/deletions (indels) in m-NHEJ-mediated genome editing. Consistently, the level of indels induced by single nicks was low and not affected by TREX2 (Figures S2B and S2C). More importantly, *Trex2* overexpression did not increase indels induced by *Sp*Cas9^H^-gWR3/gCR6 and paired *Sp*Cas9^D^-gWR3/gCL5 that generated 5′-overhanging ends but stimulated indels induced by paired *Sp*Cas9^D^-gWR3/gCR6 and paired *Sp*Cas9^H^-gWR3/gCL5 that generated 3′-overhanging ends by up to 4.7-fold and 25.1-fold, respectively (Figures S2B and S2C).

Using targeted PCR amplicon deep sequencing, we further analyzed the effect of *Trex2* overexpression on genome editing at several natural genomic sites in mouse and human cells, e.g., the *Rosa26* locus of mouse ES cells and the *HBB* and the *AAVS1* locus of human 293T cells (Figures S3A–S3C). Similarly, TREX2 did not stimulate indels induced by single *Sp*Cas9n and paired SpCas9n-sgRNAs that generated 5′-overhanging ends at these natural sites, but it generally reduced indels (Figures 1E and 1F). In contrast, *Trex2* overexpression enhanced genome editing induced by paired *Sp*Cas9^D^-gHW3/gHC4 that generated DSB ends with 30-nt 3′ overhangs at the *HBB* locus by 2.1-fold, paired *Sp*Cas9^D^-gAW1/gAC4 that generated DSB ends with 66-nt 3′ overhangs at the *AAVS1* locus by 2.4-fold, paired *Sp*Cas9^H^-gHC1/gHW3 that generated DSB ends with 65-nt 3′ overhangs at the *HBB* locus by 28.2-fold, and paired *Sp*Cas9^H^-gRW3/gRC5 that generated DSB ends with 59-nt 3′ overhangs at the *Rosa26* locus by 36.3-fold (Figures 1G and 1H). These results together suggested that *Trex2* overexpression enable paired *Sp*Cas9n-sgRNAs generating 3′-overhanging ends for efficient gene disruption.

### TREX2 promotes full or near-full deletion of 3′ overhangs generated by paired *Sp*Cas9n-sgRNAs

**Figure 2:**
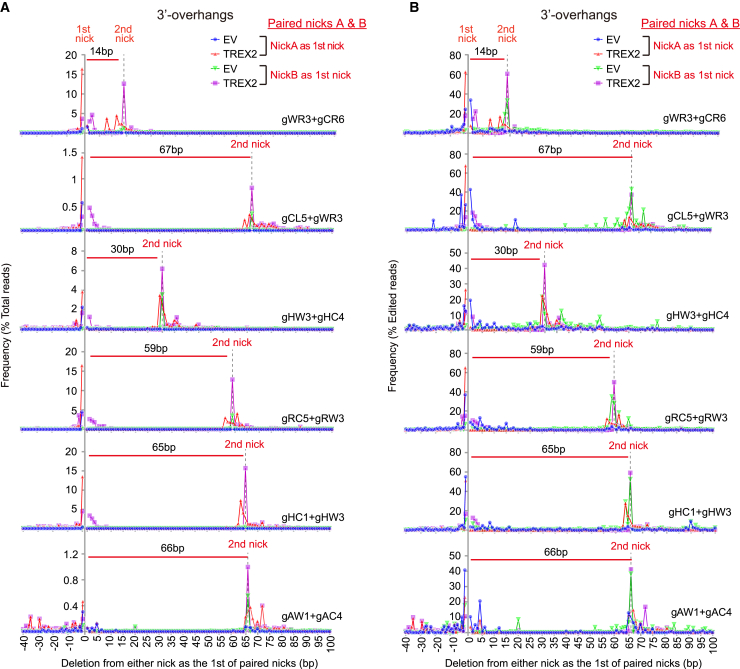
*Trex2* overexpression promotes full degradation of paired *Sp*Cas9n-induced 3′ overhangs in m-NHEJ Paired nicks (i.e., NickA and NickB) were induced on opposite strands of a specific locus by *Sp*Cas9^D^ with gWR3/gCR6, gHW3/gHC4, or gAW1/gAC4, or *Sp*Cas9^H^ with gCL5/gWR3, gRC5/gRW3, or HC1/gHW3 with or without *Trex2* overexpression, as indicated. Deletion length was defined as the distance of deletion started at either NickA as first nick or NickB as first nick at 0 bp toward or away from second nick as indicated on x axis. Deletion reads were determined by targeted amplicon Illumina sequencing and frequency of deletions with different deletion length calculated as the ratio of indicated deletion reads to total reads (A) or to edited reads (B). Either empty vector (EV) or *Trex2* overexpression for NickA as first nick and NickB as first nick is indicated by different symbols in different colors, respectively. Frequency of deletions from one nick point at 0 bp away from the other nick point is shown leftward as negative from 0 point on x axis.

To understand how overexpressed TREX2 promotes m-NHEJ of 3′-overhanging ends induced by paired *Sp*Cas9n, we analyzed the junction sequences of indels induced by *Sp*Cas9^D^-gWR3/gCR6 and *Sp*Cas9^H^-gCL5/gWR3 at the m-NHEJ reporter, *Sp*Cas9^D^-gHW3/gHC4 and *Sp*Cas9^H^-gHC1/gHW3 at the *HBB* locus, *Sp*Cas9^D^-gAW1/gAC4 at the *AAVS1* locus, and *Sp*Cas9^H^-gRC5/gRW3 at the *Rosa26* locus for the nucleotide loss from each nick site. Among total reads from targeted PCR amplicon deep sequencing, the number of indel reads for each pair of *Sp*Cas9n-sgRNA was small without *Trex2* overexpression, consistent with inefficient genome editing by paired *Sp*Cas9n generating 3′-overhanging ends (Figure 2A).^20^ Moreover, most of those indels had nucleotide loss surrounding the nick site and only a small number of them extended this nucleotide loss from the first nick (either NickA or NickB) to the site opposite to the second nick (correspondingly either NickB or NickA) (Figure 2B). In contrast, in the presence of *Trex2* overexpression, not only did indel reads increased significantly in frequency as compared to those in the absence of *Trex2* overexpression but also most of indel reads were those with full or near-full deletion of the sequences from the first nick to the site opposite to the second nick (Figures 2A and 2B). However, only a small fraction of these full deletions were precise, and *Trex2* overexpression had little effect on the portion of precise deletion among induced indels while elevating the level of indels (Figure S4).

We also analyzed the impact of overexpressed TREX2 on the junction sequences of indels induced by paired *Sp*Cas9n-sgRNAs that generate 5′-overhanging ends at the same loci. The number of indel reads among total reads for each pair of *Sp*Cas9n-sgRNAs was significant even without TREX2 overexpression as opposed to those that generate 3′-overhanging ends, consistent with efficient genome editing by paired *Sp*Cas9n generating 5′-overhanging ends (Figure S5A).^20^ Only a small portion of those indels had nucleotide loss extended from the first nick to or over the site opposite to the second nick (Figures S5A and S5B). Unlike the effect on 3′-overhanging ends, *Trex2* overexpression caused no clear alterations in the pattern of nucleotide loss for indels induced by 5′-overhanging ends (Figure S5B). These data together indicated that *Trex2* overexpression not only stimulate genome disruption induced by paired *Sp*Cas9n-sgRNAs generating 3′-overhanging ends, not 5′-overhanging ends, but also increase full loss of the intervening sequences between paired nicks, each on opposite strands inducing 3′ overhangs.

### The distance between paired nicks limits the effect of TREX2

Previous study has indicated that the maximal efficiency of paired *Sp*Cas9n generating 5′-overhanging ends in genome editing was restricted to the distance of 30–54 bp between *Sp*Cas9n-induced paired nicks.^20^ The longer distance may limit the formation or repair of 5′-overhanging ends. We tested multiple pairs of sgRNAs complexed with *Sp*Cas9^D^ or *Sp*Cas9^H^ that generated paired nicks with varying distance in the m-NHEJ reporter (Figure S2A), the *AAVS1* locus (Figure S3B), and the *hEMX1* locus (Figure S6A). Consistently, the longer the distance between *Sp*Cas9n-induced paired nicks generating 5′-overhanging ends, the lower the efficiency of genome editing these paired nicks induced (Figures S6B–S6E). In addition, we found again that *Trex2* overexpression generally reduced the efficiency of editing by paired *Sp*Cas9n-sgRNAs that generate 5′-overhanging ends (Figures S6B–S6E).

**Figure 3:**
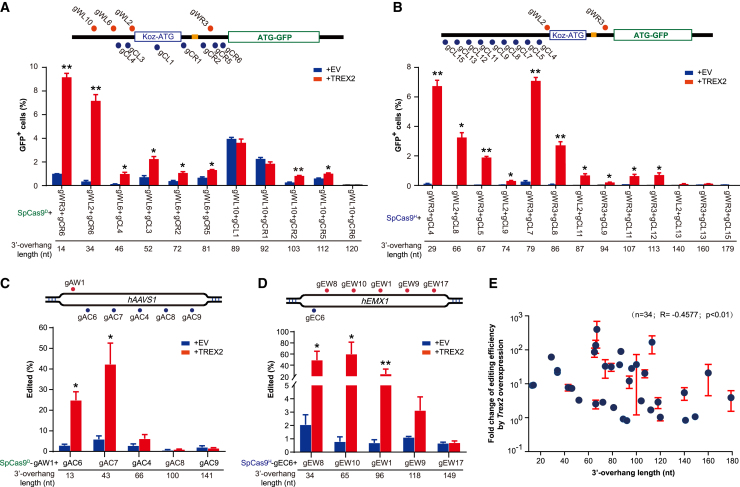
Stimulation of paired *Sp*Cas9n-induced m-NHEJ by *Trex2* overexpression was restricted to certain length of 3′ overhangs (A and B) Effect of *Trex2* overexpression on m-NHEJ of DSBs with 3′ overhangs in different length on the m-NHEJ reporter of reporter mouse ES cells. DSBs were induced by *Sp*Cas9^D^ (A) or *Sp*Cas9^H^ (B) with different sgRNA pairs as indicated. Schematic of the m-NHEJ reporter with the nicking position for each sgRNA is also shown on top. (C and D) Effect of *Trex2* overexpression on m-NHEJ of DSBs with 3′ overhangs in different length at the *hAAVS1* locus of 293T cells with *Sp*Cas9^D^ (C) or the *hEMX1* locus with *Sp*Cas9^H^ (D). sgRNA pairs for *Sp*Cas9^D^ or *Sp*Cas9^H^ are indicated. The nicking position of each sgRNA at the locus of *hAAVS1* and *hEMX1* is shown on top. Columns in (A)–(D) indicate the mean ± SEM from three independent experiments and statistics were performed by two-tailed Student’s t test. ∗p < 0.05, ∗∗p < 0.01. (E) The correlation between the length of 3′ overhangs and m-NHEJ stimulation by *Trex2* overexpression was determined by linear regression. Each blue circle indicates the mean of three independent experiments for individual paired *Sp*Cas9n-sgRNAs and the sample number (n), the correlation coefficient (R), and the probability (p) are also shown. The fold change of edited efficiency was calculated as the ratio of the edited efficiency induced by paired *Sp*Cas9n-sgRNAs in the presence of *Trex2* overexpression to that in the absence of *Trex2* overexpression (i.e., the EV control).

We further tested whether the distance between *Sp*Cas9n-induced paired nicks could affect the utility of TREX2 in genome editing by paired *Sp*Cas9n generating 3′-overhanging ends. While the editing efficiency by paired nicks that generate 3′-overhanging ends was generally negligible across the distance of 13–179 bp between these *Sp*Cas9n-induced paired nicks, significant stimulation of indel-based editing by *Trex2* overexpression appeared to be restricted to the distance of 14–81 bp between *Sp*Cas9^D^-induced paired nicks and 29–86 bp between *Sp*Cas9^H^-induced paired nicks in the m-NHEJ reporter (Figures 3A and 3B), 13–43 bp between *Sp*Cas9^D^-induced paired nicks at the *hAAVS1* locus (Figure 3C), and 34–96 bp between *Sp*Cas9^H^-induced paired nicks at the *hEMX1* locus (Figure 3D). Correlation analysis revealed that this TREX2-mediated stimulation was inversely correlated with the distance between *Sp*Cas9n-induced paired nicks generating 3′-overhanging ends (Figure 3E). Furthermore, robust editing after TREX2-mediated stimulation was observed for the distance of 13–96 bp between paired nicks (e.g., ∼9% GFP^+^ cells with the m-NHEJ reporter induced by *Sp*Cas9^D^-gWR3/gCR6 and 60% indels at the *hEMX1* locus induced by *Sp*Cas9^H^-gEC6/gEW10; Figures 3A–3D). These results indicate that the distance between *Sp*Cas9n-induced paired nicks generating 3′-overhanging ends should be taken into account when *Trex2* overexpression is used to stimulate robust genome editing by these paired nicks.

### TREX2 promotes m-NHEJ of 3′-overhanging ends at overlapping targets

**Figure 4:**
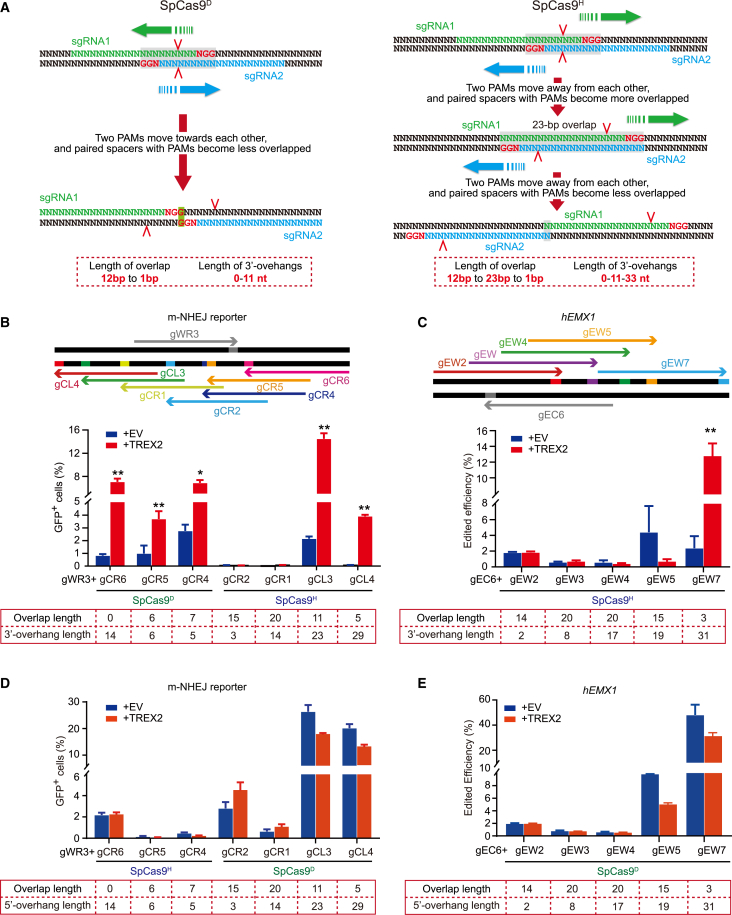
*Trex2* overexpression stimulated m-NHEJ of 3′-overhanging ends induced by paired *Sp*Cas9n on overlapping targets (A) Schematic of 3′-overhang induction by paired *Sp*Cas9^D^ (left) and *Sp*Cas9^H^ (right) on overlapping targets. Paired *Sp*Cas9^D^s generate a blunt end at a site with a 12-bp overlap. For *Sp*Cas9^D^, when paired PAMs for sgRNA1 and sgRNA2 move toward each other as indicated, the overlap length of targets starts from 12 to 1 bp, and 3′ overhangs with a length of 0–11 nt could be respectively generated by paired *Sp*Cas9^D^. For *Sp*Cas9^H^, when paired PAMs for sgRNA1 and sgRNA2 move away from each other as indicated, the overlap length of targets starts from 12 to 1 bp via 23 bp, and paired *Sp*Cas9^H^s generate 3′ overhangs with a length of 0–33 nt. The targets are indicated in green for sgRNA1 and in blue for sgRNA2, and the PAMs in red. The shaded boxes indicate the overlapping sequences and the red arrowheads on the sequences indicates the point of nicking by *Sp*Cas9n. (B–E) Effect of *Trex2* overexpression on m-NHEJ of 3′-overhanging ends and 5′-overhanging ends induced on overlapping targets by paired *Sp*Cas9^D^ or *Sp*Cas9^H^. Paired nicks that lead to DSBs with 3′ overhangs were induced on the m-NHEJ reporter in reporter mouse ES cells (B) and at the *hEMX1* locus in 293T cells (C). Paired nicks that lead to DSBs with 5′ overhangs were induced on the m-NHEJ reporter in reporter mouse ES cells (D) and at the *hEMX1* locus in 293T cells (E). Schematics of the m-NHEJ reporter and the *hEMX1* locus with the nicking position for each sgRNA as well as sgRNA pairs for *Sp*Cas9^D^ or *Sp*Cas9^H^ are also shown on top in (C) and (D). The overlap length and the overhang length are shown for each of paired *Sp*Cas9-sgRNAs under the chart. Columns in (B)–(E) indicate the mean ± SEM from three independent experiments and statistics were performed by two-tailed Student’s t test. ∗p < 0.05, ∗∗p < 0.01.

Paired *Sp*Cas9^H^-sgRNAs have been used in prime editor 3 (PE3) to improve prime editing in which the pairing of a simple sgRNA with prime editing sgRNA (pegRNA) usually targets overlapping sites.^45^ Considering that paired *Sp*Cas9n-sgRNAs are restricted to a short genomic region in targeted genome editing, extension of the method to overlapping targets may broaden its use. When the targets including the PAMs have 12-bp overlap with the PAMs close to each other, paired *Sp*Cas9n-sgRNAs generate blunt ends (Figure 4A). If the two PAMs move toward each other with the overlap being gradually reduced to 1 bp, only paired *Sp*Cas9^D^-sgRNAs could generate 3′ overhangs, which are 1–11 nt long (Figure 4A). In contrast, if the PAMs move away from each other with the overlap being first increased from 12 to 23 bp gradually and then reduced to 1 bp, only paired *Sp*Cas9^H^-sgRNAs are required to generate 3′ overhangs, which range from 1 to 33 nt in length (Figure 4A). Using the m-NHEJ reporter in mouse ES cells and the *hEMX1* locus in 293T cells, we then tested whether paired *Sp*Cas9n approach and, by extension, TREX2-mediated improvement of this approach could be used for overlapping targets. Multiple sgRNAs paired with gWR3 or gEC6 were designed to act on the opposite strands of targets that were partially or completely overlapped, inducing DSBs with 3′ overhangs in varying lengths (Figures 4B, 4C, S2A, and S6A). We found that *Trex2* overexpression stimulated m-NHEJ of 3′-overhanging ends induced by *Sp*Cas9n together with some of these overlapped sgRNA pairs (e.g., gWR3 paired with gCR5 and gCR4 for *Sp*Cas9^D^, gWR3 paired with gCL3 and gCL4, and gEC6 paired with gEW7 for *Sp*Cas9^H^; Figures 4B and 4C). The stimulation for gWR3 paired with gCR5, gCR4, gCL3, and gCL4 was even comparable to the level of the non-overlapping pair gWR3/gCR6 complexed with *Sp*Cas9^D^ (Figure 4B). As sgRNA pairs that share some or all of target sequences would be exclusive to each other for simultaneous target binding of paired *Sp*Cas9n-sgRNAs, these results suggested that paired sgRNAs could bind and nick their overlapping targets sequentially.

Due to sequential binding and nicking, overlapping *Sp*Cas9n pairs could also generate 5′-overhanging ends. As expected, m-NHEJ repair of these DSBs with 5′-overhanging ends was significant for *Sp*Cas9^D^-gWR3 paired with gCR2, gCL3, and gCL4 and *Sp*Cas9^D^-gEC6 paired with gEW5 and gEW7, but not improved by *Trex2* overexpression (Figures 4D and 4E). Interestingly, although m-NHEJ repair of 5′-overhanging ends was expected to be efficient, the editing was mostly negligible for *Sp*Cas9^H^-gWR3 paired with gCR5 or gCR4, *Sp*Cas9^D^-gWR3 paired with gCR2 or gCR1, and *Sp*Cas9^D^-gEC6 paired with gEW2, gEW3, or gEW4. For *Sp*Cas9^H^-gWR3 paired with gCR5 or gCR4, first nick was located immediately by the second PAM or within for the second *Sp*Cas9^H^-sgRNA. When the second *Sp*Cas9^H^-sgRNA unwound its target and promoted base pairing between the target strand and the spacer,^46^ it is possible that the *Sp*Cas9^H^-sgRNA-target ternary complex could not be stabilized due to the nick near or within the PAM, thus leading to inefficient induction of second nick (Figure S7A). For *Sp*Cas9^D^-gWR3 paired with gCR2 or gCR1 and *Sp*Cas9^D^-gEC6 paired with gEW2, gEW3, or gEW4, first nick was located within the target strand that serves as the nontarget strand for the second *Sp*Cas9^D^-sgRNA. Thus, when the second *Sp*Cas9^D^-sgRNA complex unwinds its target with immediate base pairing between the single-stranded target strand and the spacer of the sgRNA, the *Sp*Cas9^D^-sgRNA-target ternary complex might not be fully assembled or activated due to premature termination of the target unwinding upon encountering the nick on the nontarget strand that is no more than 17 nt away (Figure S7B). Consequently, the *Sp*Cas9^D^ nuclease with the second sgRNA could not induce a sufficient level of second nick.^46^^,^^47^

In addition, it is predicted that 3′-overhanging ends would be generated when *Sp*Cas9^H^-gWR3 was paired with gCR2 or gCR1 and *Sp*Cas9^H^-gEC6 was paired with gEW2, gEW3, or gEW4. However, the stimulatory effect of *Trex2* overexpression was not observed for these sgRNA pairs (Figures 4B and 4C). In these cases, *Sp*Cas9^H^ with the first sgRNA would nick the nontarget DNA strand at a position located within 20 nt toward the PAM for the second sgRNA (e.g., 6 and 17 nt for gWR3 paired with gCR2 and gCR1 and 5, 11, and 17 nt for gEC6 paired with gEW2, gEW3, and gEW4, respectively; Figures S7C–S7D). When *Sp*Cas9^H^ with the second sgRNA unwinds its target, the *Sp*Cas9^H^-sgRNA-target ternary complex might not be fully assembled due to premature termination of the target unwinding upon encountering first nick located on the target strand within 17 nt toward the PAM (Figures S7C and S7D). Given that at least 18 nt of the PAM-proximal target strand is required for pairing with the spacer of sgRNA in order to fully activate the nuclease activity of *Sp*Cas9n,^47^ *Sp*Cas9^H^ with the second sgRNA in these cases might not efficiently cleave its respective nontarget strand to generate DSBs with 3′ overhangs (Figures S7C and S7D). As a result, no 3′ overhangs are available for TREX2 to exert its effect.

For *Sp*Cas9^H^-gEC6/gEW5, *Sp*Cas9^H^ with the second sgRNA could fully unwind target DNA to the PAM-distal position at 20 nt due to first nick located at 22 nt away from the PAM; however, it is surprising that *Trex2* overexpression did not stimulate m-NHEJ (Figure 4C). We speculated that, upon first nick induced by *Sp*Cas9^H^, the PAM-distal 17-nt 3′-nontarget ssDNA strand released from *Sp*Cas9^H^-sgRNA might be attacked by TREX2 overexpressed (Figure S7E). Given that the pairing of at least 18 bp between the spacer of the second sgRNA and its target strand is generally required for full activation of *Sp*Cas9 nuclease,^47^ this suggests that TREX2 might degrade at least 5 nt of the PAM-distal 17-nt 3′-nontarget strand upon first nick so that only 17 nt or less are left for unwinding by *Sp*Cas9^H^ with the second sgRNA. Consequently, target unwinding by *Sp*Cas9^H^ with the second sgRNA would be prematurely terminated with no efficient induction of second nick to generate DSBs with 3′ overhangs (Figure S7E).

In contrast, in the setting of *Sp*Cas9^H^-gWR3/gCL3, first nick was located at 26 nt away from the second PAM (Figure S7F). Despite the attack on the first PAM-distal 17-nt 3′ nontarget strand by TREX2 upon first nick, m-NHEJ was still stimulated by TREX2, indicating that *Sp*Cas9^H^ with the second sgRNA could unwind target DNA to the second PAM-distal position of at least 18 nt (Figure S7F). This suggested that TREX2 could only degrade at most 8 nt of the PAM-distal 17-nt 3′-nontarget strand released from *Sp*Cas9^H^ upon *Sp*Cas9^H^-induced first nick. Together, these results defined some parameters to the rule that governs the design of overlapping sgRNA pair with *Sp*Cas9n and also suggested that the PAM-distal 17-nt 3′-nontarget strand released from *Sp*Cas9^H^ upon nicking could be attacked by the 3′-5′ exonuclease activity of TREX2 up to 8 nt. As *Trex2* overexpression enables a paired *Sp*Cas9n approach generating 3′-overhanging ends at overlapping targets for efficient gene disruption, the utility of the approach is expanded.

### *Trex2* overexpression causes little exacerbation in off-target effect of paired *Sp*Cas9n

**Figure 5:**
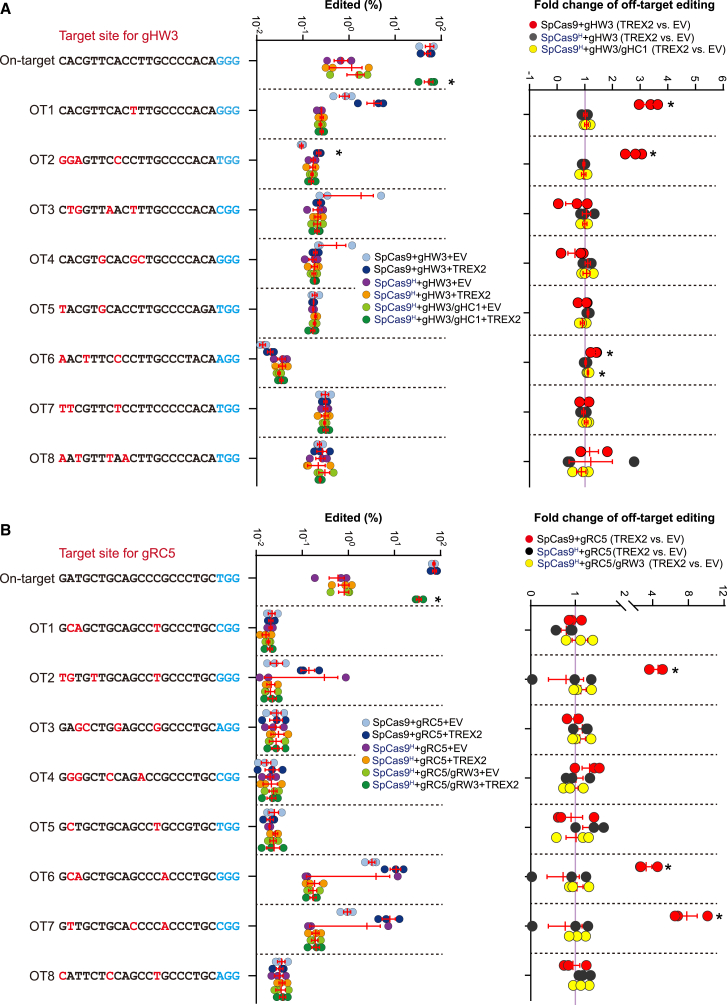
*Trex2* overexpression causes no exacerbation of off-target effect for paired *Sp*Cas9n producing 3′ overhangs Mouse ES cells were transfected with expression plasmids for *Sp*Cas9-gHW3, *Sp*Cas9^H^-gHW3, or *Sp*Cas9^H^-gHW3/gHC1 targeting the *hHBB* locus (A) or *Sp*Cas9-gRC5, *Sp*Cas9^H^-gRC5, or *Sp*Cas9^H^-gRC5/gRW3 targeting the *mRosa26* locus (B) along with expression plasmids for *Trex2* or the EV control. The indel frequencies (i.e., the percentages of the edited) at on-target and selected off-target sites for gHW3 (A) and gRC5 (B) were measured by targeted amplicon deep sequencing and defined as the ratio of edited reads to total reads normalized by transfection efficiency. Fold change of off-target editing after *Trex2* overexpression was calculated as the ratio of off-target indel frequency with *Trex2* overexpression to that with the EV control at each off-target site. Each circle indicates one independent experiment, each in triplicate, and the mean of these independent experiments is also shown. Error bars indicate SEM. Statistical significance was detected by two-tailed Student’s t test for frequencies of *Sp*Cas9-, *Sp*Cas9^H^-, or paired *Sp*Cas9^H^-induced indels between TREX2 and EV and indicated by ∗p < 0.05.

While *Trex2* overexpression enabled efficient genome editing by paired *Sp*Cas9n generating 3′-overhanging ends, we wondered whether TREX2 could increase off-target effect, which has been minimized by the use of paired *Sp*Cas9n-sgRNAs.^20^^,^^21^^,^^22^^,^^23^^,^^24^ We analyzed the frequencies of indels at on-target and eight potential off-target sites for the *hHBB*-targeting sgRNA gHW3 and for the *Rosa26*-targeting sgRNA gRC5 after genome editing at the *hHBB* locus by *Sp*Cas9-gHW3, *Sp*Cas9^H^-gHW3, as well as *Sp*Cas9^H^-gHC1/gHW3 and at the *mRosa26* locus by *Sp*Cas9-gRC5, *Sp*Cas9^H^-gRC5, as well as *Sp*Cas9^H^-gRW3/gRC5 with or without *Trex2* overexpression (Figures 5A and 5B). In the absence of *Trex2* overexpression, the on-target indel frequency induced by *Sp*Cas9-gHW3 and *Sp*Cas9-gRC5 was at least 50-fold higher than that by *Sp*Cas9^H^-gHW3 and *Sp*Cas9^H^-gHC1/gHW3 (Figure 5A). Similarly, the on-target indel frequency induced by *Sp*Cas9-gHW3 and *Sp*Cas9-gRC5 was at least 89-fold higher than that by *Sp*Cas9^H^-gRC5 and *Sp*Cas9^H^-gRW3/gRC5 (Figure 5B). However, while *Trex2* overexpression had little effect on the frequency of on-target indels induced by *Sp*Cas9 or *Sp*Cas9^H^ with a single sgRNA (either gHW3 or gRC5), the frequency of on-target indels induced by *Sp*Cas9^H^-gHC1/gHW3 and *Sp*Cas9^H^-gRW3/gRC5 was increased by ∼35- and 41-fold to the level comparable to *Sp*Cas9-gHW3 and *Sp*Cas9-gRC5, respectively (Figures 5A and 5B).

In comparison, the indel frequencies at off-target sites were low but detectable (Figures 5A and 5B). *Trex2* overexpression elevated the frequency of off-target editing by *Sp*Cas9-gHW3 at the sites of OT1, OT2, and OT6 to about 3.3-, 2.8-, and 1.4-fold and by *Sp*Cas9-gRC5 at the sites of OT2, OT6, and OT7 to about 4-, 3- and 8-fold, respectively, but had little effect at the other sites (Figures 5A and 5B). In contrast, *Trex2* overexpression did not increase off-target mutagenesis induced by *Sp*Cas9^H^ complexed with a single sgRNA or a sgRNA pair at nearly all sites tested (Figures 5A and 5B). These results together indicated that TREX2 overexpression caused no additional off-target effect while enabling efficient genome editing by paired *Sp*Cas9n generating 3′-overhanging ends.

### XRCC4 is required for TREX2-mediated stimulation in m-NHEJ of 3′-overhanging ends

Previous study has demonstrated that endogenous TREX2 was not required for local m-NHEJ of I-SceI-induced DSBs.^37^ While *Trex2* overexpression improved m-NHEJ of 3′-overhanging ends induced by paired *Sp*Cas9n-sgRNAs, it was unclear whether endogenous TREX2 was required for this repair. We thus deleted endogenous *Trex2* in m-NHEJ reporter mouse ES cells and found that loss of *Trex2* did not affect the level of I-SceI-induced NHEJ by comparing seven *Trex2*^*−/−*^ clones with three isogenic *Trex2*^*+/+*^ clones (Figures S8A and S8B). Deletion of *Trex2* also had little effect on m-NHEJ of blunt ends, 3′-overhanging ends, and 5′-overhanging ends induced by *Sp*Cas9, *Sp*Cas9^D^, and *Sp*Cas9^H^ in complex with gWR3/gCR6, respectively (Figure S8C). However, *Trex2* overexpression in *Trex2*^*−/−*^ cells as well as in *Trex2*^*+/+*^ cells still increased m-NHEJ of blunt ends and 3′-overhanging ends respectively induced by *Sp*Cas9-gWR3/gCR6 and *Sp*Cas9^D^-gWR3/gCR6, but had no effect on m-NHEJ of 5′-overhanging ends induced by *Sp*Cas9^H^-gWR3/gCR6 (Figure S8C). This suggested that m-NHEJ of 3′-overhanging ends is not controlled by endogenous TREX2 in cells but could be promoted by TREX2 exogenously overexpressed.

**Figure 6:**
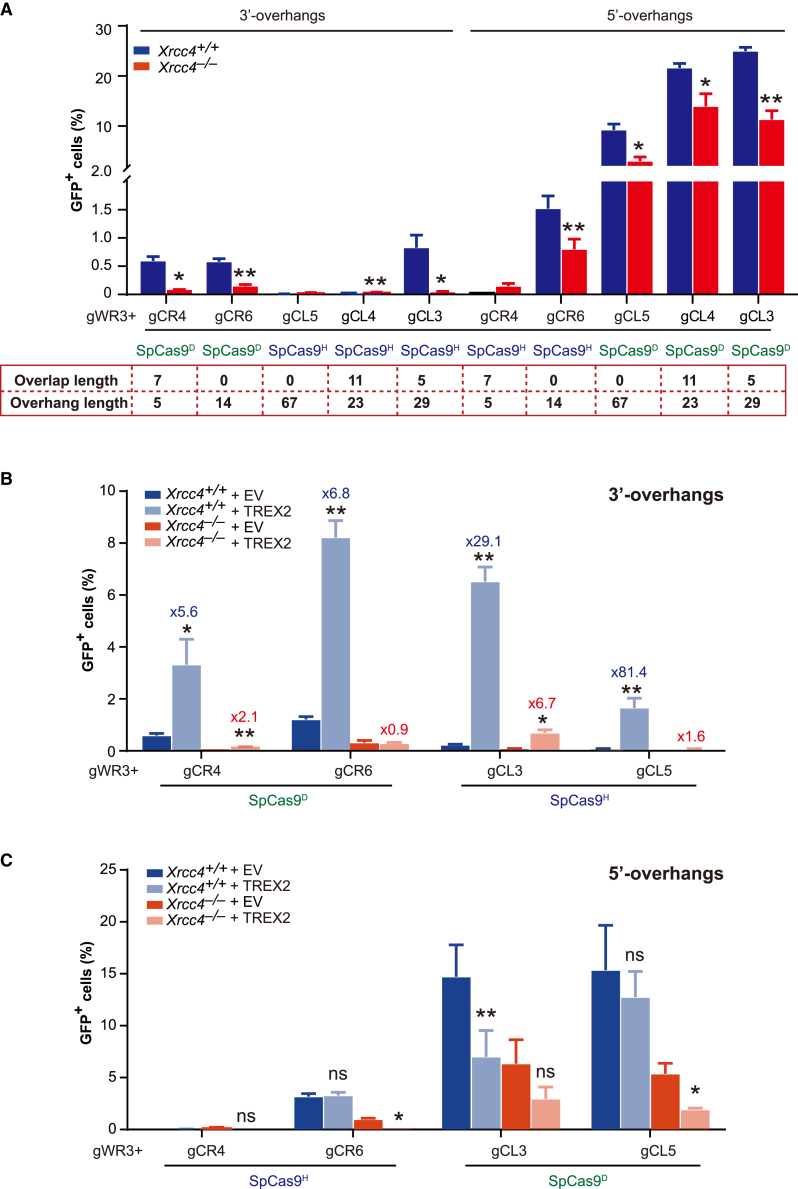
*Xrcc4* deletion reduced stimulation of m-NHEJ by *Trex2* overexpression (A) Effect of *Xrcc4* deletion on m-NHEJ of DSBs induced by paired *Sp*Cas9n-sgRNAs on overlapping and non-overlapping targets of the m-NHEJ reporter in reporter mouse ES cells. (B and C) Effect of *Xrcc4* deletion on m-NHEJ stimulation by *Trex2* overexpression. DSBs with 3′-overhanging ends (B) or 5′-overhanging ends (C) were induced as indicated by paired *Sp*Cas9n-sgRNAs on overlapping and non-overlapping targets of the m-NHEJ reporter in *Xrcc4*^*+/+*^ and *Xrcc4*^*−/−*^ reporter mouse ES cells. *Sp*Cas9n-gWR3/gCR4 and *Sp*Cas9n-gWR3/gCL3 targeted overlapping sites and *Sp*Cas9n-gWR3/gCR6 and *Sp*Cas9n-gWR3/gCL5 targeted non-overlapping sites. The fold of stimulation by *Trex2* overexpression is indicated above each column in (B). Columns indicate the mean ± SEM from three independent experiments, each in triplicate, and statistics were performed by two-tailed Student’s t test. ∗p < 0.05; ∗∗p < 0.01; ns, not significant.

Previous studies have shown that the c-NHEJ factor XRCC4 is required for efficient NHEJ of I-SceI- or *Sp*Cas9-induced DSBs processed by TREX2.^37^^,^^38^ Unlike I-SceI and *Sp*Cas9, which respectively generate DSB ends with 4-nt 3′ overhangs and blunt ends, paired *Sp*Cas9n-sgRNAs produce DSB ends with 5′ or 3′ overhangs in varying lengths, some of which are usually long. As the polarity and length of end overhangs may affect the engagement of DSB ends to c-NHEJ in yeast and class switch recombination (CSR) in mouse and human B cells,^48^^,^^49^^,^^50^ we wondered whether XRCC4 was required for m-NHEJ repair of DSB ends with 5′ overhangs and 3′ overhangs of varying length in mouse ES cells. Using isogenic *Xrcc4*^*+/+*^ and *Xrcc4*^*−/−*^ NHEJ reporter mouse ES cells previously established,^7^ we found that deletion of *Xrcc4* reduced m-NHEJ of overhanging ends induced by paired *Sp*Cas9n-sgRNAs generating 14-, 23-, 29-, or 67-nt 5′ overhangs and 3′ overhangs at non-overlapping and overlapping targets (Figure 6A). This suggested that XRCC4 was also required for efficient m-NHEJ of DSB ends with long 5′ overhangs and 3′ overhangs in mouse ES cells. However, despite the reduction in the absence of XRCC4, this m-NHEJ repair remained robust for DSBs with 5′-overhanging ends even in *Xrcc4*^*−/−*^ cells. Similarly, activation-induced cytidine deaminase (AID) could generate paired, staggered nicks on immunoglobin loci for CSR,^51^ which is also supported by robust *Xrcc4*-independent a-EJ.^52^ While *Trex2* overexpression dramatically stimulated m-NHEJ of 3′-overhanging ends induced by paired *Sp*Cas9n-sgRNAs in *Xrcc4*^*+/+*^ mouse ES cells, this TREX2-mediated stimulation was significantly reduced from 5.6-, 29.1-, and 81.4-fold to 2.1-, 6.7-, and 1.6-fold, respectively, for *Sp*Cas9^D^-WR3/CR4, *Sp*Cas9^H^-WR3/CL3, and *Sp*Cas9^H^-WR3/CL5 or even abolished for *Sp*Cas9^D^-WR3/CR6 in *Xrcc4*^*−/−*^ mouse ES cells (Figure 6B). This indicated that XRCC4 promotes full stimulation of m-NHEJ of 3′-overhanging ends by TREX2, but the underlying mechanism is yet to be determined. In contrast, TREX2 either reduced or had little effect on m-NHEJ of 5′-overhanging ends in both *Xrcc4*^*+/+*^ and *Xrcc4*^*−/−*^ mouse ES cells (Figure 6C).

### Fusion of TREX2 to *Sp*Cas9n improves the utility of paired *Sp*Cas9n strategy

**Figure 7:**
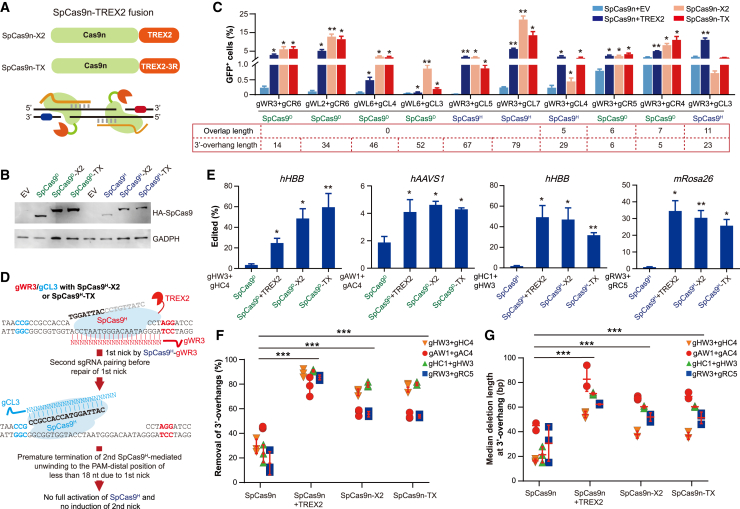
TREX2 fused to *Sp*Cas9n promoted m-NHEJ of 3′-overhanging ends induced by paired *Sp*Cas9n-sgRNAs (A) Schematic for *Sp*Cas9n-TREX2 fusion. The TREX2 and its variant with R163A, R165A, and R167A mutations were fused to the C termini of *Sp*Cas9n to generate the *Sp*Cas9n-X2 and *Sp*Cas9n-TX, respectively. (B) Steady-state level of *Sp*Cas9n-TREX2 fusion proteins as well as *Sp*Cas9n alone. Mouse ES cells were transfected with expression plasmids indicated and harvested 3 days post transfection for western blot. (C) Effect of TREX2 fusion on m-NHEJ of 3′-overhanging ends induced by paired *Sp*Cas9n. Mouse ES cells containing the m-NHEJ reporter were transfected with expression plasmids for paired *Sp*Cas9n-X2 or paired *Sp*Cas9n-TX as well as for paired *Sp*Cas9n together with either the EV control or expression plasmids for *Trex2*. DSBs with 3′-overhanging ends were induced on non-overlapping and overlapping targets of the m-NHEJ reporter and repair of DSBs by m-NHEJ was measured as the frequency of GFP^+^ cells by FACS 3 days post transfection. Paired sgRNAs with *Sp*Cas9^D^ and *Sp*Cas9^H^ are shown on x axis. The overlap length and 3′-overhang length are also indicated under the chart for each of paired *Sp*Cas9n-sgRNAs. (D) Model explaining the inability of paired gWR3/gCL3 with *Sp*Cas9^H^-X2 or *Sp*Cas9^H^-TX to induce a DSB at an overlapping target. Upon first nick by *Sp*Cas9^H^ with gWR3 as an example, fused TREX2 could degrade more than 8 nt of the PAM-distal 17-nt 3′-nontarget strand released from the *Sp*Cas9^H^-gWR3-target ternary complex. Before repair of first nick, only 17 nt or less are left for target DNA unwinding by *Sp*Cas9^H^ with gCL3. Consequently, the *Sp*Cas9^H^-gCL3-target ternary complex could not be fully assembled or stabilized to induce second nick and few DSBs generated for genome editing. (E) Effect of TREX2 fusion on m-NHEJ of 3′-overhanging ends induced by paired *Sp*Cas9n at the loci of *hHBB* and *hAAVS1* in 293T cells and the *mRosa26* locus of mouse ES cells. Paired sgRNAs, i.e., gHW3/gHC4, gAW1/gAC4, gHC1/gHW3, and gRW3/gRC5, with *Sp*Cas9^D^ and *Sp*Cas9^H^ are indicated on x axis. (F) Removal of 3′ overhangs by TREX2 fused to *Sp*Cas9n. Percentage of 3′-overhang removal was determined by analysis of the junctions at four natural genomic sites targeted by paired *Sp*Cas9n, paired *Sp*Cas9n with TREX2 overexpressed, paired *Sp*Cas9n-X2, and paired *Sp*Cas9n-TX along with paired sgRNAs indicated. (G) Effect of TREX2 fused to *Sp*Cas9n on median deletion length in m-NHEJ of 3′-overhanging ends induced by paired *Sp*Cas9n-TREX2. Median deletion length was determined by junction analysis at four natural genomic sites targeted by *Sp*Cas9n, *Sp*Cas9n together with TREX2 overexpressed, *Sp*Cas9n-X2, and *Sp*Cas9n-TX, in combination with paired sgRNAs indicated. Columns in (C) and (E) indicate the mean ± SEM from three independent experiments, each in triplicate, and statistics were performed by two-tailed Student’s t test. In (F) and (G), each symbol represents one independent experiment and statistical significance was detected by one-way ANOVA for increased median deletion length by TREX2 fusion as well as *Trex2* overexpression. ∗p < 0.05, ∗∗p < 0.01, ∗∗∗p < 0.001.

To simplify the coupling of *Trex2* overexpression in paired *Sp*Cas9n-sgRNAs generating 3′-overhanging ends, we fused hemagglutinin (HA)-tagged *Sp*Cas9n with TREX2 to generate *Sp*Cas9n-X2 (Figure 7A). We also fused HA-tagged *Sp*Cas9n with the DNA-binding-deficient mutant of TREX2 (i.e., TREX2-3R carrying R163A, R165A, and R167A mutations) to generate *Sp*Cas9n-TX as a safer alternative because these mutations are expected to minimize non-specific DNA contact.^12^ The steady-state levels of HA-tagged *Sp*Cas9n-X2 and *Sp*Cas9n-TX proteins were similar to those of HA-tagged *Sp*Cas9n at 3 days after transfection into m-NHEJ reporter mouse ES cells (Figure 7B). As compared to *Trex2* overexpression, both *Sp*Cas9n-X2 and *Sp*Cas9n-TX induced a comparable or even higher level of m-NHEJ that repairs complementary 3′-overhanging ends for nearly all sgRNA pairs tested except gWR3/gCL3 (Figures 7C and S9A). For *Sp*Cas9^H^-gWR3/gCL3, TREX2-mediated stimulation of m-NHEJ indicated that nucleotide degradation by TREX2 could reach, at most, 8 nt of the PAM-distal 17-nt 3′-nontarget strand released from *Sp*Cas9^H^-sgRNA upon *Sp*Cas9^H^-induced first nick located at 26 nt away from the second PAM, consistent with the data in Figure S7F and supplemental information. In contrast, because no stimulatory effect of *Sp*Cas9^H^-X2 or *Sp*Cas9^H^-TX on m-NHEJ was detected (Figure 7C), it is possible that *Sp*Cas9^H^-X2 and *Sp*Cas9^H^-TX degrade more than 8 nt of the PAM-distal 17-nt 3′-nontarget strand upon first nick so that only 17 nt or less were left for target unwinding by *Sp*Cas9^H^-X2 and *Sp*Cas9^H^-TX with the second sgRNA (Figure 7D). Consequently, *Sp*Cas9^H^-X2 and *Sp*Cas9^H^-TX with the second sgRNA would not be sufficiently activated to induce second nick for DSB generation.

We also compared genome editing by paired *Sp*Cas9n, *Sp*Cas9n coupled with TREX2 overexpression, *Sp*Cas9n-X2, and *Sp*Cas9n-TX, each pair generating 3′-overhanging ends respectively at four natural genomic sites, including two *hHBB* targets, one *hAAVS1* target, and one m*Rosa26* target. In complex with any of four sgRNA pairs, while *Sp*Cas9n induced insignificant genome editing as expected, *Sp*Cas9n-X2 and *Sp*Cas9n-TX increased the efficiency of genome editing to ∼2- to 35-fold as did comparably *Sp*Cas9n coupled with *Trex2* overexpression (Figures 7E and S9B). Like TREX2 overexpression that promoted full or near-full deletion of 3′ overhangs generated by paired *Sp*Cas9n-sgRNAs, both paired *Sp*Cas9n-X2 and *Sp*Cas9n-TX also induced full or near-full deletion of 3′ overhangs at these four natural sites (Figure 7F). In addition, with each pair of sgRNAs tested, *Sp*Cas9n coupled with TREX2 overexpression, *Sp*Cas9n-X2, and *Sp*Cas9n-TX induced indels with increased median deletion length as compared to *Sp*Cas9n alone (Figure 7G). These results together indicated that fusion of wild-type TREX2 or the safer mutant TREX2-3R to *Sp*Cas9n not only simplify the approach of paired *Sp*Cas9n-sgRNAs coupled with *Trex2* overexpression at no expense of genome editing efficiency but also improve the utility of this strategy with easier use and potentially better safety.

## Discussion

Off-target effect is a serious problem in CRISPR-Cas9 genome editing, and many strategies have been implemented to address this safety concern.^15^^,^^16^^,^^17^^,^^18^^,^^19^^,^^20^ Among the strategies, double nicking on opposite strands by paired Cas9 nickases was devised to minimize off-target effects while maintaining efficient genome editing and has been useful in many applications.^3^^,^^20^^,^^25^^,^^26^^,^^27^^,^^28^ However, this approach was usually only efficient with paired Cas9^D^ nickases generating 5′-overhanging ends, not with paired Cas9^D^ or Cas9^H^ nickases generating 3′-overhanging ends or with paired Cas9^H^ nickases generating 5′-overhanging ends.^20^^,^^21^^,^^22^^,^^23^ In this study, we found that ectopic overexpression of *Trex2* or TREX2 fusion to *Sp*Cas9n significantly elevated the efficiency of genome disruption by paired *Sp*Cas9n generating 3′-overhanging ends while retaining minimal off-target effect, enabling paired *Sp*Cas9n generating 3′-overhanging ends in genome editing. Fusion of TREX2 to *Sp*Cas9n provided a simplified but potentially safer version of the approach. Although previous studies have demonstrated that ectopic expression of TREX2 or *Sp*Cas9-TREX2 fusion did not affect cell proliferation or survival,^12^^,^^34^^,^^40^ the safety concern of using TREX2 in genome editing is further alleviated by the fusion of DNA-binding-deficient TREX2 mutant to *Sp*Cas9n, which is expected to minimize the global activity of TREX2 ectopically expressed.^12^^,^^39^

The improvement of paired *Sp*Cas9n-sgRNAs by coupled *Trex2* overexpression or by *Sp*Cas9n-TREX2 fusion could offer an additional window for efficient genome editing with reduced off-target effect to target sequences where paired *Sp*Cas9^D^s do not generate 5′-overhanging ends. Furthermore, while the paired-*Sp*Cas9^H^ approach is little used due to low efficiency as compared to paired *Sp*Cas9^D^, *Trex2* overexpression and TREX2 fusion could significantly increase the efficiency of genome editing by paired *Sp*Cas9^H^ generating 3′-overhanging ends at many sites to a level comparable to or even better than those induced by *Sp*Cas9 and paired *Sp*Cas9^D^, allowing paired *Sp*Cas9^H^ to be a useful alternative in a toolbox of genome editing. In addition, paired nicks on opposite strands by *Sp*Cas9n can mimic certain types of endogenous DNA damage, e.g., AID-induced DNA damage in CSR, in order to elucidate the mechanisms underlying repair of this type of DNA damage and advance our understanding in relevant fields. In fact, paired nicks induced by *Sp*Cas9n each on opposite strands were employed in mouse and human B cells to mimic AID-induced nicks and were able to induce CSR.^49^^,^^50^ This application has led to further understanding of CSR in antibody diversification.^49^^,^^50^^,^^53^^,^^54^ Since DSBs with 5′ overhangs induced CSR more efficiently than DSBs with 3′ overhangs,^49^^,^^50^ TREX2 may help promote CSR induced by 3′-overhanging ends and allow a further, previously ignored, study in this regard.

Paired *Sp*Cas9n-sgRNA has also been employed to improve prime editing in addition to its canonical applications in minimizing off-target effects.^17^ In PE3, paired *Sp*Cas9^H^-RT was used to induce double nicks, among which one by pegRNA primes reverse transcription to generate the edited strand and the other by a simple sgRNA directs its repair using the edited strand as the template.^45^ Among sites targeted by paired *Sp*Cas9^H^-RT, some are overlapped but exhibit a high level of prime editing, and some others have poor editing efficiency. Even in efficient prime editing, indels induced by paired *Sp*Cas9^H^-RT pose a serious problem. Recent development of paired prime editors that involve paired nicks on opposite strands in a long distance has led to more efficient prime editing and allows prime editing with precise deletion and insertion of large DNA sequences.^17^^,^^41^^,^^42^^,^^43^^,^^44^ It is expected that these paired nicks on opposite strands have a chance to generate a DSB with 5′- or 3′-overhanging ends. Therefore, understanding the mechanisms underlying the conversion of paired nicks on opposite strands into a DSB could yield some insight into a potential solution to enhance prime editing and minimize indels. This effort could be assisted by TREX2, which may help interrogate strand resection and strand separation started at the sites of paired nicks. Consequently, as an example of potential applications, a proper control on end resection or unwinding from nicks on non-edited strands could improve the pairing of edited strand and loss of non-edited strands in prime editing that generates precise deletion and insertion of a large DNA sequence. Prime editing may also be helped by more time for reverse transcription before DSB generation if we could extend target binding and residence of *Sp*Cas9^H^-RT at the nick for reverse transcription, not for repair of the nicked, complementary strand.^7^^,^^55^

It is unclear how TREX2 degrades 3′ overhangs in genome editing by paired *Sp*Cas9n-sgRNAs. However, free TREX2 or TREX2 fusion with *Sp*Cas9n could act on at least three types of 3′-ssDNA substrates during genome editing by paired *Sp*Cas9n-sgRNAs. The first one is the PAM-distal 17-nt 3′-nontarget strand released from *Sp*Cas9^H^ upon *Sp*Cas9^H^-mediated nicking (Figure S10A). It is well established that *Sp*Cas9 remains tightly bound to its target even after cleavage but locally releases the PAM-distal 17-nt 3′-nontarget strand.^46^ This 17-nt 3′-nontarget strand can be accessed and chewed away by TREX2 overexpressed and TREX2 fused to *Sp*Cas9^H^. However, due to immediate proximity of TREX2 upon target cleavage by *Sp*Cas9^H^-TREX2, *Sp*Cas9^H^-TREX2 could degrade at least 9 nt of 17-nt 3′-nontarget strand, whereas free TREX2 only degrades 5–8 nt of the same 17-nt 3′-nontarget strand, assuming that PAM-proximal 18-bp pairing between the spacer and target strand is required to fully activate *Sp*Cas9.^47^ Such extended degradation by TREX2 fused to *Sp*Cas9^H^ may be further explored for additional applications, such as investigation of end resection at a nick. In addition, although both *Sp*Cas9^D^ and *Sp*Cas9^H^ can generate 3′-overhanging ends, which are sensitive to TREX2-mediated degradation, no PAM-distal 17-nt 3′-nontarget strand is released from *Sp*Cas9^D^-induced nick. Therefore, it appears that *Sp*Cas9^H^-induced 3′ overhangs are more sensitive to TREX2 than *Sp*Cas9^D^-induced 3′ overhangs. In *Sp*Cas9- and *Sa*Cas9-induced DSBs, degradation of the PAM-distal 17-nt 3′-nontarget strand by TREX2 could create a 5′-overhanging end and a blunt end of the DSBs, thus converting accurate NHEJ of original blunt ends into m-NHEJ between the 5′-overhanging end and the blunt end and increasing indel-based genome editing. This helps explain why *Trex2* overexpression stimulated m-NHEJ induced by *Sp*Cas9 and *Sa*Cas9 but not by *Lb*Cas12a, which generates no 3′-ssDNA strand.^56^^,^^57^

Second, 3′-ssDNA released by BLM-mediated 3′-5′ unwinding from paired nicks can serve as a substrate for TREX2 (Figure S10B). Paired nicks on opposite strands were generally thought to produce a staggered DSB with the overhang length equal to the distance that separates paired nicks.^20^^,^^25^^,^^49^ However, as nicks separated by long distance are not expected to spontaneously melt into a staggered DSB, helicases and nucleases in cells may be recruited and activated by paired nicks, thus converting these nicks into a staggered DSB with varying overhang lengths. Previous study indicated that BLM could bind to a nick and track onto 3′-end to unwind DNA from 3′ to 5′, releasing 3′-ssDNA.^58^ This 3′-ssDNA could be attacked by TREX2 during the process of unwinding and/or after unwinding. However, in paired nicks induced by *Sp*Cas9^H^, TREX2 could degrade PAM-distal 17-nt 3′-nontarget strands and create a gap at the target sites after dissociation of *Sp*Cas9^H^-sgRNA from its targets. Although the gap might also recruit helicases and nucleases to induce end resection and unwinding, generating a DSB with overhanging ends, it is yet to be determined whether the mechanism underlying gap-induced end resection and unwinding are different from nick-induced end resection and unwinding.

The third type of substrate is 3′-overhanging ends. Given the function of Mre11 and BLM in short-range end resection, paired nicks could be simultaneously degraded from 3′ to 5′ by Mre11 to generate DSB ends with 3′ overhangs,^59^ which provide a substrate for free TREX2 and *Sp*Cas9n-TREX2 (Figure S10C). Because both paired *Sp*Cas9^D^-sgRNAs and paired *Sp*Cas9^H^-sgRNAs can generate 3′-overhanging ends, free TREX2 and *Sp*Cas9n-TREX2 remain effective in stimulating m-NHEJ repair of 3′-overhanging ends via TREX2-mediated degradation. However, in the approach of paired *Sp*Cas9^H^-sgRNAs targeting overlapping sites, it appears that TREX2-mediated degradation of the PAM-distal 17-nt 3′-nontarget strand at the first nick occur first to efficiently block the complete and stable assembly of the second *Sp*Cas9^H^-sgRNA to its target, although target-bound *Sp*Cas9^H^-sgRNA may interfere with TREX2-mediated degradation. Consequently, the second nick is not efficiently induced or even not induced at all.

Repair of DNA breaks in mammalian cells requires nucleases, helicases, and polymerases and their regulatory factors to resect, unwind, and synthesize DNA. Many of these enzymes and regulatory factors have been used in combination to improve or expand CRISPR genome editing.^3^^,^^17^ However, while PE3 and paired prime editors have combined reverse transcriptase with paired *Sp*Cas9^H^ that induce nicks on opposite strands in prime editing with small edits and large deletions or insertions,^3^^,^^17^ few have been explored to modify paired *Sp*Cas9n for better efficiency and specificity in SSB- or DSB-based genome editing. As this study shows that TREX2 can be harnessed to enable efficient genome editing by paired *Sp*Cas9n generating 3′-overhanging ends, it is of interest to test other nucleases, helicases, polymerases, and their regulatory factors that, by either co-expression or fusion to *Sp*Cas9n, may act at nicks during end resection, DNA unwinding, and DNA synthesis during genome editing by paired *Sp*Cas9n generating 5′- or 3′-overhanging ends to improve the paired *Sp*Cas9n approach in genome editing and broaden its applications.

## Materials and methods

### Plasmids

The CRISPR-Cas9 plasmid px330 was originally obtained from Addgene (Catalog no. #42230). Plasmids expressing HA-tagged *Sp*Cas9n (*Sp*Cas9^D^ and *Sp*Cas9^H^) were generated by site-directed mutation KOD Plus-Neo Kit and were subcloned in to pcDNA-3β-Hyg vector.^60^ The expression plasmids for I-SceI, *Staphylococcus aureus* Cas9 (*Sa*Cas9), and *Lachnospiraceae bacterium* Cas12a (*Lb*Cas12a) were described previously.^7^ Expression plasmids of sgRNAs were generated from the U6-sgRNA vector (pU6-gRNA) as described before.^6^ The sgRNA target sequences are listed in Table S2. Full-length mouse *Trex2* was amplified by PCR with the primer pair mTREX2-F and mTREX2-R from a mouse ES cell cDNA library and subcloned into pcDNA-3β-Hyg vector to generate a *Trex2* expression plasmid (Table S3). The TREX2 and DNA-binding-deficient TREX2 mutant TREX2-3R (i.e., R163A, R165A, and R167A) were amplified with the primer pair TREX2-F1 and TREX2-R1 respectively from *Sp*Cas9-X2 and *Sp*Cas9-TX plasmids (a gift from Jiazhi Hu at Peking University)^12^ and fused to the C terminus of *Sp*Cas9^D^ and *Sp*Cas9^H^ to generate expression plasmids for Cas9^D^-X2, Cas9^D^-TX, Cas9^H^-X2, and Cas9^H^-TX (Table S3). Plasmids newly constructed were confirmed by Sanger sequencing.

### Cell lines

The m-NHEJ reporter mouse ES cells were previously established and cultured as described before.^7^ Isogenic *Xrcc4*^*+/+*^ and *Xrcc4*^−/−^ mouse ES cells containing the m-NHEJ reporter were previously generated.^7^ Human embryonic kidney 293T (HEK293T) cells were cultured in high-glucose DMEM containing 10% fetal bovine serum, 1% penicillin-streptomycin, and 2 mM L-glutamine. For *Sp*Cas9-mediated *Trex2* knockout in cells containing m-NHEJ reporter, 2 × 10^5^ mouse ES cells were transfected with the expression plasmid for paired sgRNAs and *Sp*Cas9 and were seeded on mouse embryonic fibroblast (MEF) feeder cells for single clones without any antibiotics screening.^61^ Knockout clones were verified by PCR along with Sanger sequencing. Primers are listed on Table S3.

### Western blot and antibodies

Cells were harvested 72 h post transfection, washed by cold PBS, and lysed with RIPA buffer for 30 min. Cell extractions were separated by SDS-PAGE electrophoresis and the proteins were transferred to polyvinylidene fluoride (PVDF) membrane (Millipore). The membrane was incubated with primary antibody at 4°C for 8 h and then incubated with secondary antibody at 24°C for 1 h. The proteins were detected by Bio-Rad chemiluminescence imager. The primary antibodies used in this study were mouse monoclonal anti-HA probe (SC-7392; 1:1,000) from Santa Cruz and mouse monoclonal anti-GAPDH (EM1101; 1:5,000) from HuaBio.

### Transfection and m-NHEJ reporter assays

Transfection of mouse ES cells was performed with Lipofectamine 2000 (Invitrogen) in 24-well plates as previously described.^6^^,^^61^ A total of 2 × 10^5^ mouse ES cells harboring the m-NHEJ reporter were transfected with 0.5 μg of I-SceI, *Sa*Cas9-sgRNA, *Lb*Cas12a-sgRNA, *Sp*Cas9-sgRNA, or *Sp*Cas9n-sgRNA by 1.2 μL of Lipofectamine 2000. In TREX2 overexpression assays, cells were co-transfected with 0.2 μg of *mTrex2* expression plasmid and the expression plasmids for 0.15 μg of *Sp*Cas9n plasmid and 0.15 μg of sgRNA plasmid. For HEK293T cells, 1.0 × 10^5^ cells were seeded on a 24-well plate and grown to 80%–95% confluence. A total of 1.0 μg of DNAs were transfected by 2.4 μL of Lipofectamine 2000. In *Trex2* overexpression assays, 0.4 μg of *Trex2* expression plasmid were transfected along with 0.3 μg of *Sp*Cas9n and 0.3 μg of sgRNA plasmids.

Transfected cells were analyzed for GFP^+^ frequencies using the Beckman Coulter CytoFLEX flow cytometer after 72 h post transfection. The m-NHEJ frequencies were calculated after being corrected with background readings and normalized with transfection efficiencies as described before.^7^^,^^10^ Statistical comparisons between two unpaired populations and between paired samples were analyzed by one-way ANOVA or Student’s two-tailed paired t test, respectively.

### Genomic DNA extraction and PCR amplification

For genome editing at m-NHEJ reporter or endogenous locus, expression plasmids for I-SceI, *Sp*Cas9, *Sp*Cas9n, *Sa*Cas9, or *Lb*Cas12a were transfected with sgRNA. At 60–72 h post transfection, cells were harvested and genomic DNA was isolated for analysis of genome editing. Genomic DNA was isolated from these cells using a genomic DNA purification kit (Vazyme). The target regions were PCR amplified, with respective primers listed in Table S3.

### Targeted amplicon deep sequencing

The target regions were PCR-amplified with respective primers listed in Table S3. PCR products were purified using a PCR Clean-up kit (Vazyme). The PCR amplicon was generated according to the manufacturer’s protocols (Yeasen, Hieff NGS Ultima DNA Library Prep Kit for Illumina) and next-generation sequencing was performed at Novogene. Sequences were analyzed to identify edited events with different indels at repair junctions using DBS-Aligner as described previously.^6^

### Off-target analysis

Potential off-target sites were identified using the latest version of the CRISPR off-target prediction website (http://crispor.tefor.net/). All potential sites were ranked by an off-target hit score, and high-ranked potential sites were selected. Off-target sites were amplified by PCR with primers listed in Table S3. On-target and off-target editing efficiencies were determined by Illumina deep sequencing and calculated as indel frequencies.^7^ The fold change of off-target frequency is calculated as the ratio of indel frequency without *Trex2* overexpression to indel frequency with *Trex2* overexpression at each off-target site.

## Data and code availability

Deep-sequencing raw data are available in the Sequence Read Archive (SRA) under accession number PRJNA861435 (https://www.ncbi.nlm.nih.gov/sra/PRJNA861435). Flow cytometry raw data for this study have also been deposited at Zenodo, where it is directly accessible at https://doi.org/10.5281/zenodo.7181264 and https://doi.org/10.5281/zenodo.7192218.

## Supplemental information

Document S1. Figures S1–S10 and Tables S2 and S3

Table S1. Source data This Excel file contains the source data for Figures 1–7 and Figures S1–S4 and S6–S9.

Document S2. Article plus supplemental information
