# Supplementary material for: TREX2 enables efficient genome disruption mediated by paired CRISPR-Cas9 nickases that generate 3′-overhanging ends
Source: Mol Ther Nucleic Acids. 2023 Nov 2;34:102072. doi: 10.1016/j.omtn.2023.102072 (PMC10661556; doi:10.1016/j.omtn.2023.102072)
Supplement: Document S1. Figures S1–S10 and Tables S2 and S3 [file mmc1.pdf]

OMTN, Volume *34*

Supplemental information

TREX2 enables efficient genome disruption mediated by paired CRISPR-Cas9 nickases that generate 3′-overhanging ends

Yue Wang, Yi-Li Feng, Qian Liu, Jing-Jing Xiao, Si-Cheng Liu, Zhi-Cheng Huang, and An-Yong Xie

Table S1. Source data. This Excel file contains the source data for Figure 1-7 and Supplementary Figure S1-S4 and S6-S9.

Table S2. List of sgRNAs used in this study

sgRNA	Sequence (5'-3')	PAM (5'-3')	RNA-bound Strand	Length (bp)
gCL1	GATAACAGGGTAATCCATGG	TGG	W	20
gWL2	TTACCCTAACCGCCGCCACCA	TGG	C	21
gCL3	AGGGTAATCCATGGTGGCGG	CGG	W	20
gCL4	ATCCATGGTGGCGGCGGTTA	GGG	W	20
gCL5	TTGAGCTCGAGATCTGAGTC	CGG	W	20
gWL6	CCTGCAGCCCAAGCTCTAGC	CGG	C	20
gCL7	TCTGAGTCCGGCTAGAGCTT	GGG	W	20
gCL8	CCGGCTAGAGCTTGGGCTGC	AGG	W	20
gCL9	AGCTTGGGCTGCAGGTCGAA	AGG	W	20
gWL10	TTCTCCTCTTCCTCATCTCC	GGG	C	20
gCL11	GGTCGAAAGGCCCGGAGATG	AGG	W	20
gCL12	AAGGCCCGGAGATGAGGAAG	AGG	W	20
gCL13	TTTGAAGCGTGCAGAATGCC	GGG	W	20
gCL15	CGGGCCTCGGGAGGACCTTC	GGG	W	20
gCR1	AGGGATAACAGGGTAATCCA	TGG	W	20
gCR2	GGATGGATCCTAGGGATAAC	AGG	W	20
gWR3	TGGATTACCCTGTTATCCCT	AGG	C	20
gCR4	CATGTGAAGGATGGATCCTA	GGG	W	20
gCR5	TCATGTGAAGGATGGATCCT	AGG	W	20
gCR6	CTTGCTGATCATGTGAAGGA	TGG	W	20
gRW3	CCTAAGAATGAGAAAGGCAA	AGG	C	20
gRC5	GATGCTGCAGCCCGCCCTGC	TGG	W	20
gHC1	GGTATCAAGGTTACAAGAC	AGG	W	19
gHW3	CACGTTCACCTTGCCCCACA	GGG	C	20
gHC4	CATGGTGCATCTGACTCCTG	AGG	W	20
gAW1	GTCCCCTCCACCCCACAGT	GGG	C	19
gAC4	AGACCCAATATCAGGAGACT	AGG	W	20
gAC6	GTCACCAATCCTGTCCCTAG	TGG	W	20
gAC7	AAGGAGGAGGCCTAAGGATG	GGG	W	20
gAC8	AGATAAGGAATCTGCCTAAC	AGG	W	20
gAC9	GCAAGGAGAGAGATGGCTCC	AGG	W	20
gEW1	GAGTCCGAGCAGAAGAAGAA	GGG	C	20
gEW2	CATCTGTGCCCCTCCCTCCC	TGG	C	20

gEW3	TGCCCCTCCCTCCCTGGCCC	AGG	C	20
gEW4	TCCCTCCCTGGCCCAGGTGA	AGG	C	20
gEW5	CCCTGGCCCAGGTGAAGGTG	TGG	C	20
gEC6	TCACCTGGGCCAGGGAGGGA	GGG	W	20
gEW7	TGAAGGTGTGGTTCCAGAAC	CGG	C	20
gEW8	AGGTGTGGTTCCAGAACCGG	AGG	C	20
gEW9	GCTCCCATCACATCAACCGG	TGG	C	20
gEW10	CAAACGGCAGAAGCTGGAGG	AGG	C	20
gEW17	CACGAAGCAGGCCAATGGGG	AGG	C	20
ga2	CCCTGTTATCCCTAGGATCC ATC	ATTA	C	23
g1	AGGATGGATCCTAGGGATAA	CAGGGT	W	20
TREX2-KO-gRNA2	TTCCTGGACCTAGAAGCCAC	TGG	C	20
TREX2-KO-gRNA6	CAGAACACGGGGCAGCACCA	AGG	W	20

Table S3. List of PCR primers used in this study

PCR primers	Sequence (5'-3')
**ON-Target primers**	
mRosa26-F	ATGAGTTGCTGGTGAAGACG
mRosa26-R	GAATCCGTGCAAGCCAAG
mTREX2-KO-F	CTGAGATGGCTATAGGTAGC
mTREX2-KO-R	TTGGACCATCAGGTGGCACG
NHEJ-reporter system-F1	CCGCGCTGTTCTCCTCTTC
NHEJ-reporter system-R1	ATCGCCCTCGCCCTCGCCGG
NHEJ-reporter system-F2	TCCCGAGGCCCGGCATTCTG
NHEJ-reporter system-R2	GCCGTCCAGCTCGACCAGGA
hHBB-F	GGGTGGGAAAATAGACCAAT
hHBB-R	AGAGCCATCTATTGCTTAC
hAAVS1-F1	TGGGACCACCTTATATTCCC
hAAVS1-R1	TCTAAGGTTTGCTTACGATG
hAAVS1-Overlapping-F2	TCCCAGGGCCGGTTAATGTG
hAAVS1-Overlapping-R2	GGCTCCATCGTAAGCAAACC
hAAVS1-Distal-F3	GGGCCGGTTAATGTGGCTCT
hAAVS1-Distal-R3	TCCCAGGATCCTCTCTGGCT
hEMX1-Overlapping-F1	CTCAGTCTTCCCATCAGGCTC
hEMX1-Overlapping-R1	GATGTGATGGGAGCCCTTCTT
hEMX1-Distal-F2	CTCAGTCTTCCCATCAGGCTC
hEMX1-Distal-R2	GAGGTGACATCGATGTCCTC
hEMX1-Distal-F3	CTGAGTGTTGAGGCCCCAGT
hEMX1-Distal-R3	AGCAAGCAGCACTCTGCCCT
**OFF-Target primers**	
mRosa26-OT1-F	GGTTAGAGGGAGGGTGGGAG
mRosa26-OT1-R	TCATCAACAGGCAGCAATCC
mRosa26-OT2-F	TCTACCACTGGCCTGTTCAT
mRosa26-OT2-R	AGGCACAGATGGGCTGTAGA
mRosa26-OT3-F	AAAAAGGAACTTGAATAAAA
mRosa26-OT3-R	GCATATCATTGTCTTAGGAA
mRosa26-OT4-F	CAAGCCTCACCTAGAAACCG
mRosa26-OT4-R	CTGGACTCAGGCACGAGGAG
mRosa26-OT5-F	CAATAGAGAAACACCTGCCT
mRosa26-OT5-R	TGTTGAAATGTTGTCTCAGTA
mRosa26-OT6-F	AACCTGGTAGTTGTCACTGC
mRosa26-OT6-R	TCCTCCAGTGCTTGTTTCTGC
mRosa26-OT7-F	AGAAAGAGGCGGAGCTAGAG

mRosa26-OT7-R	CAGTAGCAACACAGACCTCG
mRosa26-OT8-F	AGGGAGTCTGCTGGGGGACAG
mRosa26-OT8-R	CTCCCTTTGGTGTGTGAGGA
HBB-OT1-F	CAGTAATCTGAGGGTAGGAA
HBB-OT1-R	ACACTTTCTTCTGACATAAC
HBB-OT2-F	GGCAGCAGACAAGGCAGGAA
HBB-OT2-R	CAGCACCATGTTTAGCGTCC
HBB-OT3-F	AGTGAGCGTAGGCTCTGACA
HBB-OT3-R	AACGCCCAGGGAGATGCAGC
HBB-OT4-F	ATCCTCTAAGAGTGACCTCA
HBB-OT4-R	GCCAGAGGGAGAGAAATCTG
HBB-OT5-F	TCAGTAGGACAGGGCAGAAG
HBB-OT5-R	CTATGTTAGAAAGTAGTGCC
HBB-OT6-F	ATGAATCCTGGCTATGGGAG
HBB-OT6-R	AGGATTTGCCTTCCCTGAAC
HBB-OT7-F	GAATCCAGGTAAGAGATTAT
HBB-OT7-R	TGGCACTAGATGGGCCTGGA
HBB-OT8-F	ATTATAGACATAAGCCGCTA
HBB-OT8-R	TATCTCATGAGAAGTCTACTC
**Plasmid construction primers**	
mTREX2-F	CTCGGATCCCATATGTCTGAGCCACCCCGGGCT
mTREX2-R	ATTCTAGATTATCAGGCTTCGAGGCTTGGACCATCA
TREX2-F1	GCCAGGCAAAAAAGAAAAAGGGAGGAGGCGGCAGCATGTC
TREX2-R1	GTTTAAACGGGCCCTCTAGATTAGGCCTCCAGGCTGGGGT

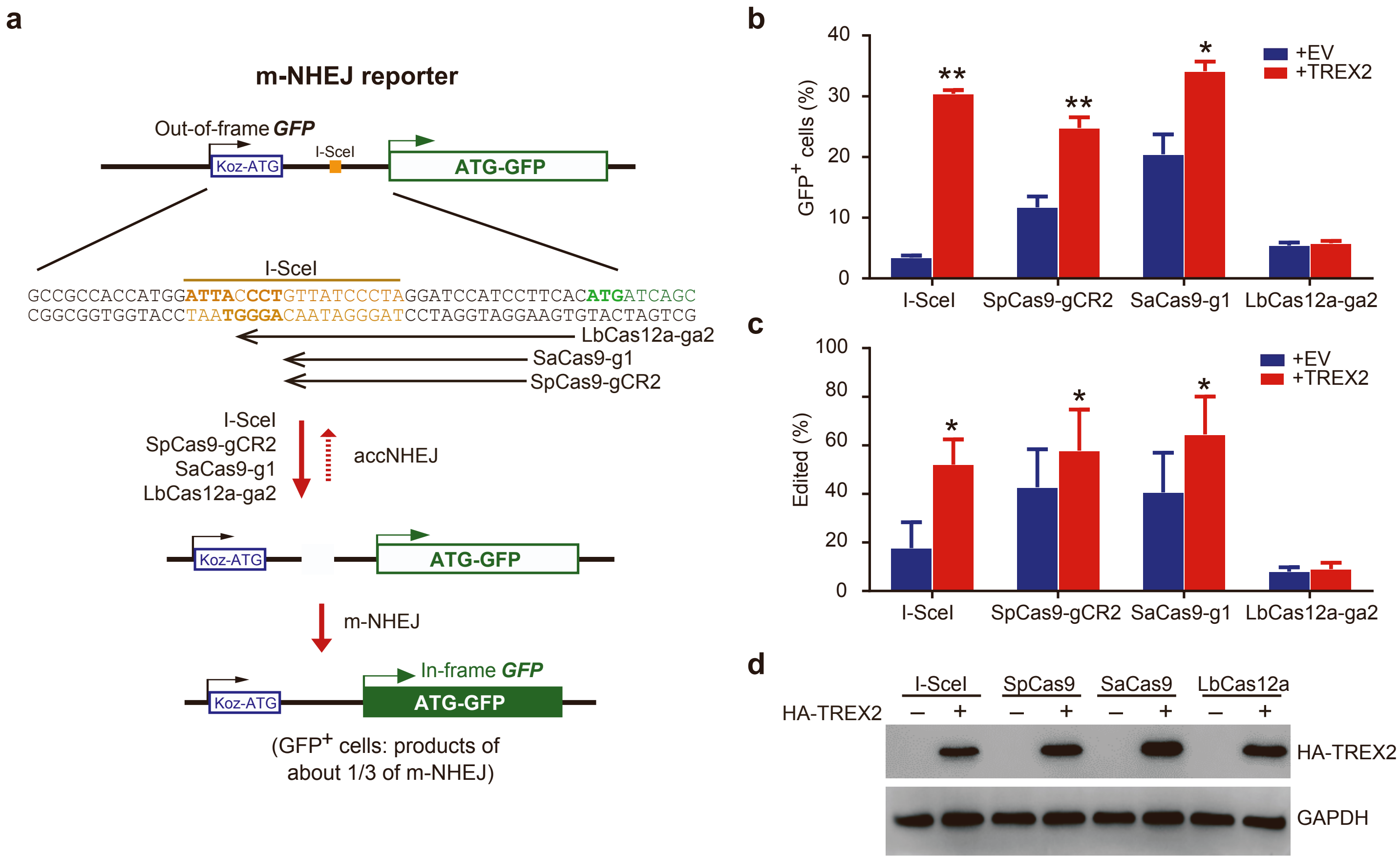

Figure S1. *Trex2* overexpression exerted different effect on m-NHEJ of DSBs induced by I-SceI, *Sp*Cas9, *Sa*Cas9 and *Lb*Cas12a. (**a**) Schematic of sgRNAs and target sequences in the m-NHEJ reporter. Target sequences are indicated by arrowed lines, and the PAM sequence labeled in bold. accNHEJ: accurate NHEJ. (**b, c**) Effect of *Trex2* overexpression on I-SceI-, *Sp*Cas9-, *Sa*Cas9- and *Lb*Cas12a-induced m-NHEJ. Mouse ES cells containing the m-NHEJ reporter were transfected with indicated plasmids and harvested 3 days post transfection for measurement of GFP^+ cells by FACS (**b**) and for the frequency of edited by targeted amplicon Illumina deep sequencing (**c**). (**d**) Steady-state level of HA-tagged TREX2 overexpressed in reporter mouse ES cells along with expression of I-SceI, *Sp*Cas9, *Sa*Cas9 or *Lb*Cas12a. GAPDH was the loading control for Western blot.

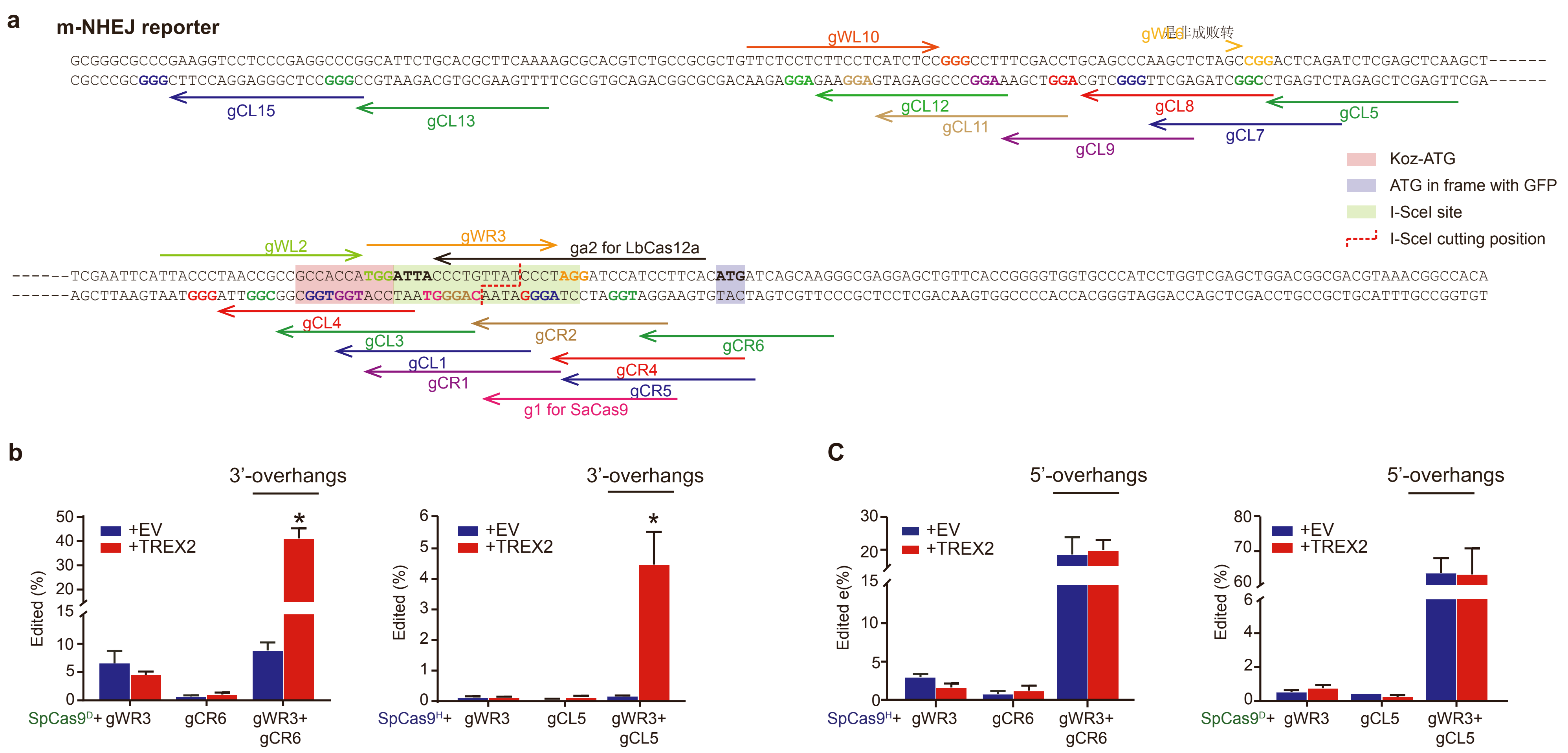

Figure S2. *Trex2* overexpression stimulated the m-NHEJ of 3'-overhanging ends generated by paired *Sp*Cas9n. (**a**) Target sequences in the m-NHEJ reporter by paired sgRNAs with *Sp*Cas9n. Target sequences by sgRNAs are indicated by arrowed lines, and the PAM sequences are labeled in bold. Koz-ATG, the start codon ATG for *GFP*, the I-SceI site and I-SceI cutting position are also shown. (**b, c**) Effect of *Trex2* over-expression on m-NHEJ induced by single or paired *Sp*Cas9n. Mouse ES cells containing the m-NHEJ reporter were transfected with expression plasmids for single or paired *Sp*Cas9^{D}-sgRNAs and *Sp*Cas9^{H}-sgRNAs along with the EV vector or expression plasmids for *Trex2*. Paired *Sp*Cas9^{D}-sgRNAs and *Sp*Cas9^{H}-sgRNAs expressed generated 3'-overhanging ends (**b**) and 5'-overhanging ends (**c**), respectively. Genomic DNA was harvested 3 days post transfection and the edited efficiency was determined by targeted amplicon Illumina deep sequencing.

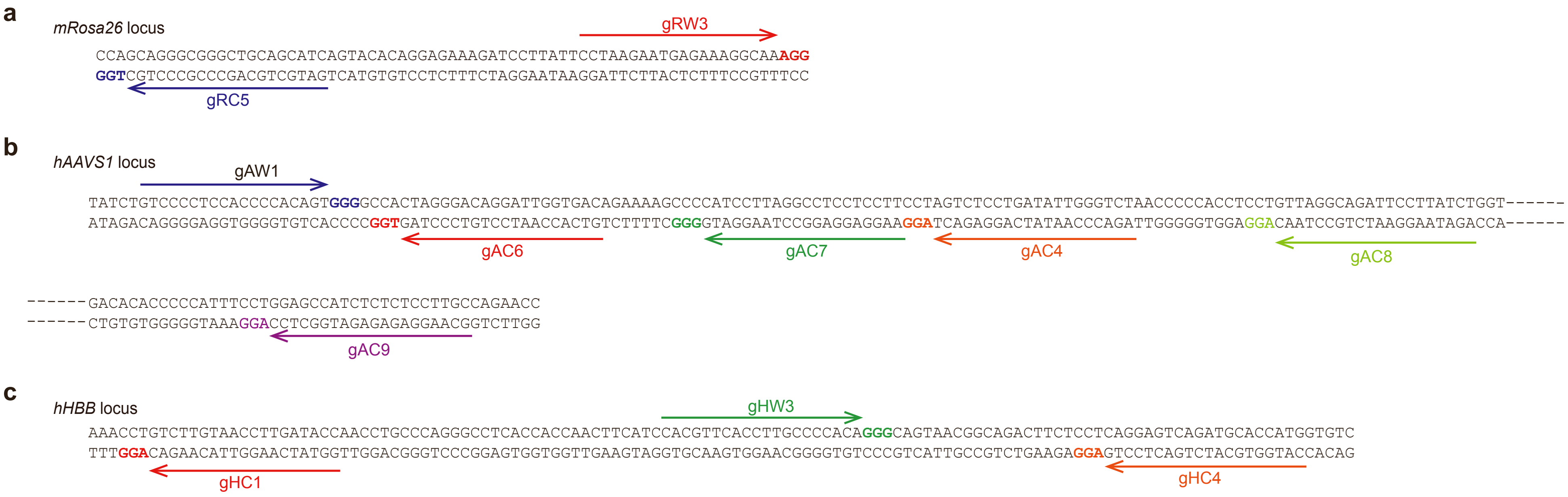

Figure S3. Target sequences in the loci of m*Rosa26* (a), h*AAVS1* (b) and h*HBB* (c) by single or paired sgRNAs with *Sp*Cas9n. Target sequences by sgRNAs are indicated by arrowed lines, and the PAM sequences are labeled in color.

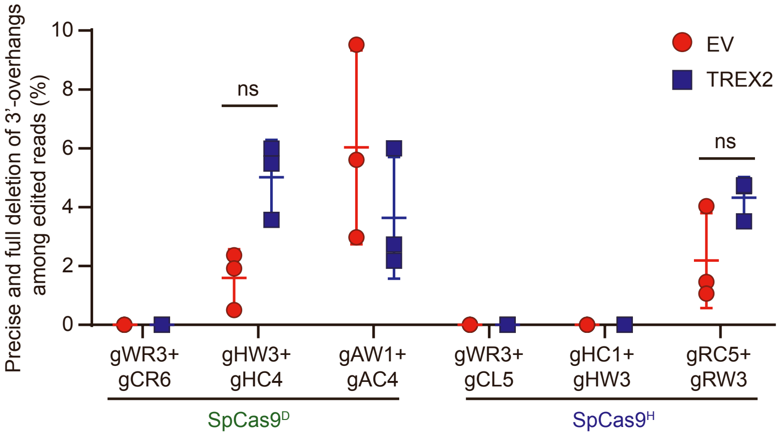

Figure S4. *Trex2* overexpression had little effect on precise and full deletion of 3'-overhangs in m-NHEJ repair of DSBs induced by paired *Sp*Cas9n. Junction of m-NHEJ with or without *Trex2* overexpression was analyzed by targeted amplicon Illumina deep sequencing. The frequency of precise and full deletion of 3'-overhangs among edited reads was calculated as the ratio of reads for precise and full deletion of 3'-overhangs to edited reads. Each symbol represents one independent experiment in triplicates, and the mean of three independent experiments is also indicated. Error bars indicate S.E.M.

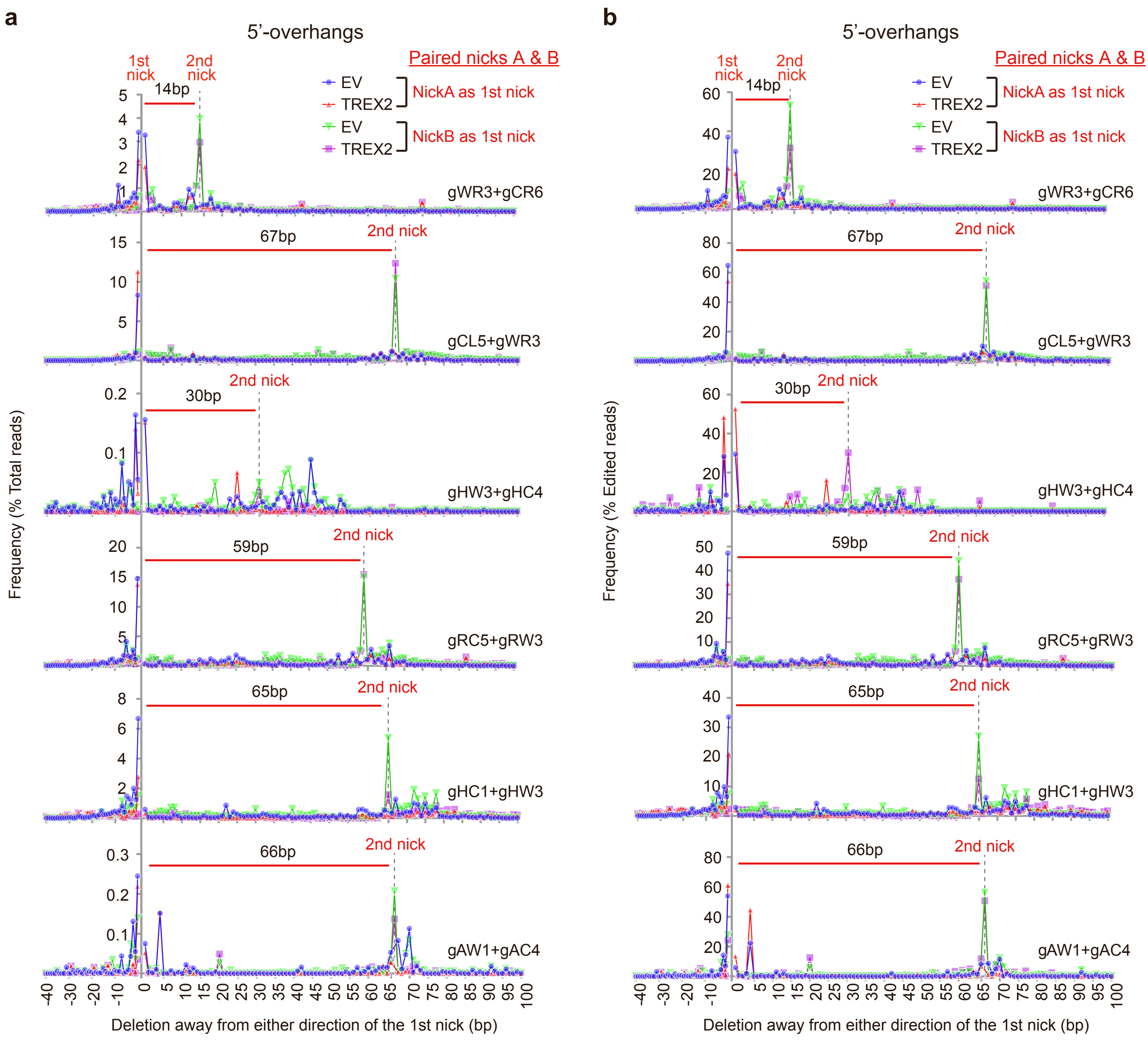

Figure S5. ***Trex2* overexpression had minimal effect on deletion of 5'-overhangs in m-NHEJ of DSBs induced by paired *Sp*Cas9n.** Paired nicks (i.e., NickA and NickB) were induced on opposite strands of a specific locus to generate a DSB with 5'-overhangs by *Sp*Cas9^{H} with gWR3/gCR6, gHW3/gHC4 or gAW1/gAC4, or *Sp*Cas9^{D} with gCL5/gWR3, gRC5/gRW3 or HC1/gHW3 with or without *Trex2* overexpression as indicated. Deletion length was defined as the distance of deletion started at either NickA as 1st nick or NickB as 1st nick at "0 bp" towards or away from 2nd nick as indicated on X-axis. Deletion reads were determined by targeted amplicon Illumina sequencing and frequency of deletions with different deletion length calculated as the ratio of indicated deletion reads to total reads (**a**) or to edited reads (**b**). Either EV or *Trex2* overexpression for NickA as 1st nick and NickB as 1st nick is indicated by different symbols in different colors, respectively. Frequency of deletions from one nick point at "0 bp" away from the other nick point is shown leftwards as negative from '0' point on X-axis.

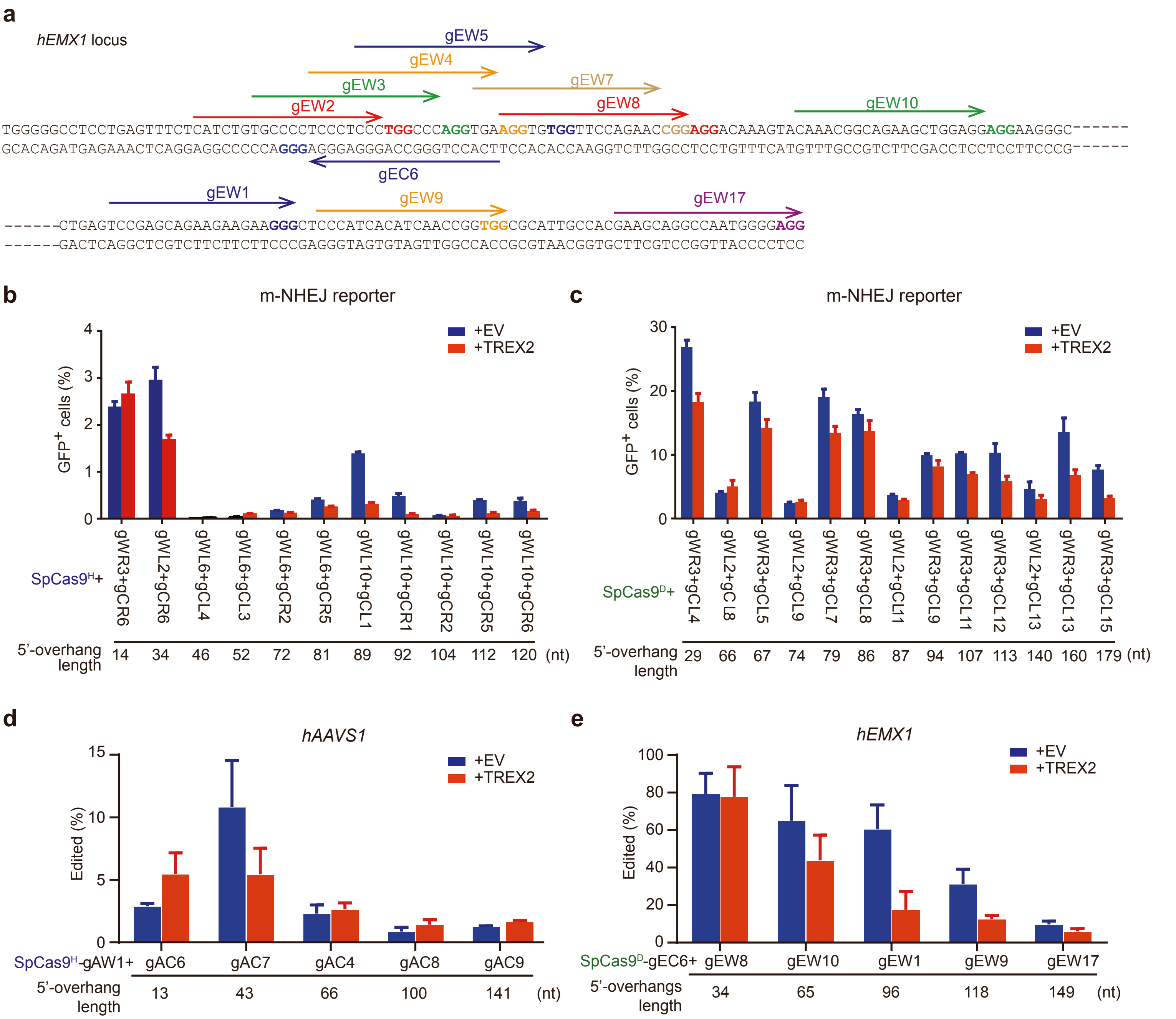

Figure S6. *Trex2* overexpression caused no stimulation of m-NHEJ in repair of paired *Sp*Cas9n-induced DSBs with 5'-overhangs in different length. (**a**) Target sequences in the *hEMX1* locus by *Sp*Cas9n-sgRNAs. Target sites by sgRNAs are indicated by arrowed lines, and the PAM sequences are labeled in color. (**b, c**) Effect of *Trex2* overexpression on m-NHEJ of DSBs with 5'-overhangs in different length on the m-NHEJ reporter of reporter mouse ES cells. DSBs were induced by *Sp*Cas9^{H} (**b**) or *Sp*Cas9^{D} (**c**) with different sgRNA pairs as indicated. 5'-overhang length is also shown under the chart. (**d, e**) Effect of *Trex2* overexpression on m-NHEJ of DSBs with 5'-overhangs in different length at the *hAAVS1* locus of 293T cells with *Sp*Cas9^{H} (**d**) or the *hEMX1* locus with *Sp*Cas9^{D} (**e**). sgRNA pairs for *Sp*Cas9^{D} or *Sp*Cas9^{H} are indicated. The length of 5'-overhangs is also indicated under the chart. Columns in b-e indicate the mean ± S.E.M from three independent experiments.

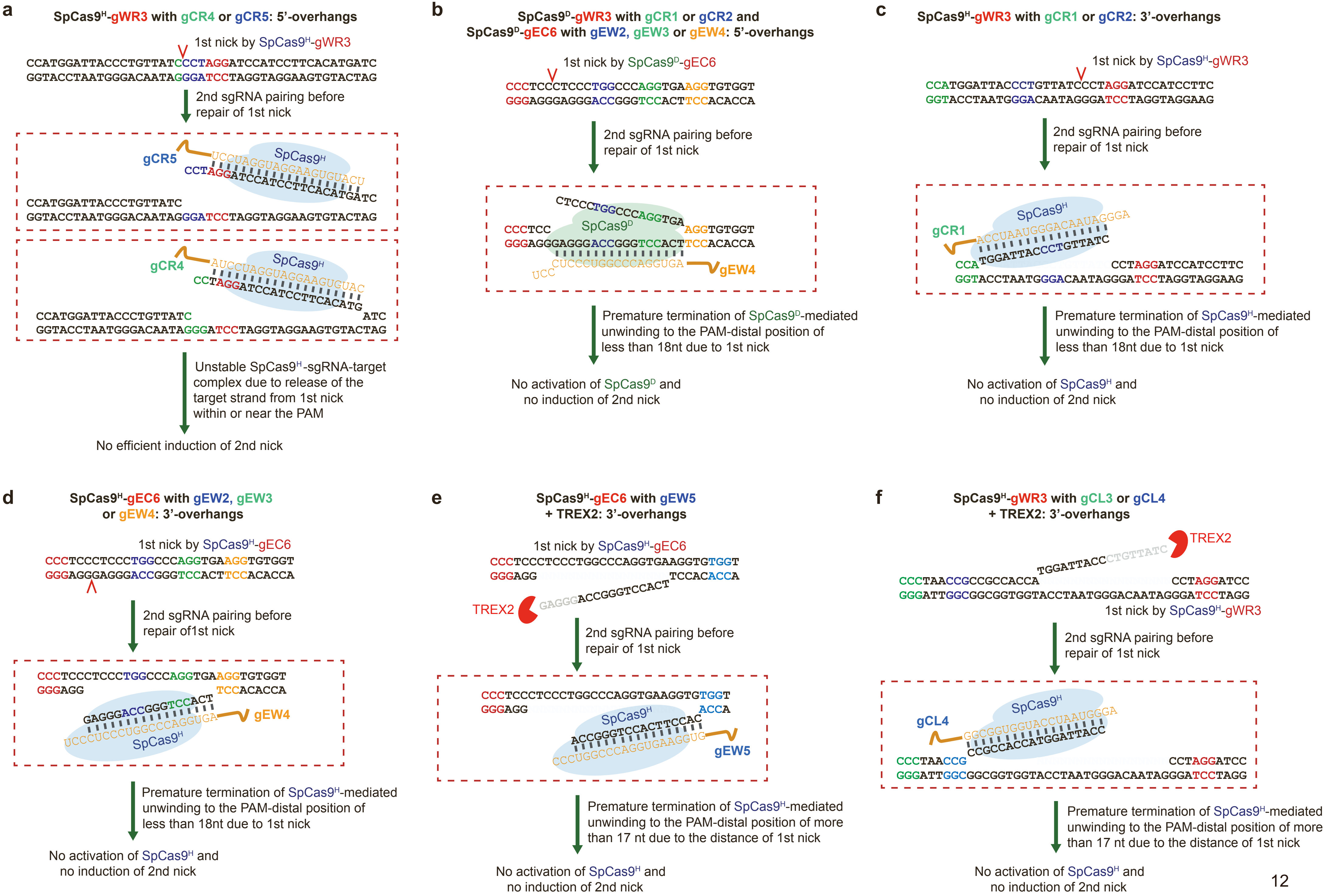
a
SpCas9H-gWR3 with gCR4 or gCR5: 5'-overhangs
1st nick by SpCas9H-gWR3
CCATGGATTACCCTGTTATCCCTAGGATCCATCCTTCACATGATC
GGTACCTAATGGGACAATAGGGATCCTAGGTAGGAAGTGTACTAG
2nd sgRNA pairing before repair of 1st nick
gCR5
SpCas9H
UCCUAGGUAGGAAGUGUACU
CCTAGGATCCATCCTTCACATGATC
CCATGGATTACCCTGTTATC
GGTACCTAATGGGACAATAGGGATCCTAGGTAGGAAGTGTACTAG
gCR4
SpCas9H
AUCCUAGGUAGGAAGUGUAC
CCTAGGATCCATCCTTCACATG
CCATGGATTACCCTGTTATC
ATC
GGTACCTAATGGGACAATAGGGATCCTAGGTAGGAAGTGTACTAG
Unstable SpCas9H-sgRNA-target complex due to release of the target strand from 1st nick within or near the PAM
No efficient induction of 2nd nick
b
SpCas9D-gWR3 with gCR1 or gCR2 and SpCas9D-gEC6 with gEW2, gEW3 or gEW4: 5'-overhangs
1st nick by SpCas9D-gEC6
CCCTCCCTCCCTGGCCCAGGTGAAGGTGTGGT
GGGAGGGAGGGACCGGGTCCACTTCCACACCA
2nd sgRNA pairing before repair of 1st nick
CTCCCTGGCCCAGGTGA
SpCas9D
CCCTCC
AGGTGTGGT
GGGAGGGAGGGACCGGGTCCACTTCCACACCA
UCC
CUCCCUGGCCCAGGUGA
gEW4
Premature termination of SpCas9D-mediated unwinding to the PAM-distal position of less than 18nt due to 1st nick
No activation of SpCas9D and no induction of 2nd nick
c
SpCas9H-gWR3 with gCR1 or gCR2: 3'-overhangs
1st nick by SpCas9H-gWR3
CCATGGATTACCCTGTTATCCCTAGGATCCATCCTTC
GGTACCTAATGGGACAATAGGGATCCTAGGTAGGAAG
2nd sgRNA pairing before repair of 1st nick
SpCas9H
gCR1
ACCUAAUGGGACAAUAGGGA
CCA
TGGATTACCCTGTTATC
CCTAGGATCCATCCTTC
GGTACCTAATGGGACAATAGGGATCCTAGGTAGGAAG
Premature termination of SpCas9H-mediated unwinding to the PAM-distal position of less than 18nt due to 1st nick
No activation of SpCas9H and no induction of 2nd nick
d
SpCas9H-gEC6 with gEW2, gEW3 or gEW4: 3'-overhangs
1st nick by SpCas9H-gEC6
CCCTCCCTCCCTGGCCCAGGTGAAGGTGTGGT
GGGAGGGAGGGACCGGGTCCACTTCCACACCA
2nd sgRNA pairing before repair of1st nick
CCCTCCCTCCCTGGCCCAGGTGAAGGTGTGGT
GGGAGG
TCCACACCA
GAGGGACCGGGTCCACT
UCCCUCCCUGGCCCAGGUGA
gEW4
SpCas9H
Premature termination of SpCas9H-mediated unwinding to the PAM-distal position of less than 18nt due to 1st nick
No activation of SpCas9H and no induction of 2nd nick
e
SpCas9H-gEC6 with gEW5 + TREX2: 3'-overhangs
1st nick by SpCas9H-gEC6
CCCTCCCTCCCTGGCCCAGGTGAAGGTGTGGT
GGGAGG
TCCACACCA
TREX2
GAGGGACCGGGTCCACT
2nd sgRNA pairing before repair of 1st nick
CCCTCCCTCCCTGGCCCAGGTGAAGGTGTGGT
GGGAGG
ACCA
SpCas9H
ACCGGGTCCACTTCCAC
CCCUGGCCCAGGUGAAGGUG
gEW5
Premature termination of SpCas9H-mediated unwinding to the PAM-distal position of more than 17 nt due to the distance of 1st nick
No activation of SpCas9H and no induction of 2nd nick
f
SpCas9H-gWR3 with gCL3 or gCL4 + TREX2: 3'-overhangs
TREX2
TGGATTACCCTGTTATC
CCCTAACCGCCGCCACCA
CCTAGGATCC
GGGATTGGCGGCGGTGGTACCTAATGGGACAATAGGGATCCTAGG
1st nick by SpCas9H-gWR3
2nd sgRNA pairing before repair of 1st nick
SpCas9H
gCL4
GGCGGUGGUACCUAAUGGGA
CCGCCACCATGGATTACC
CCCTAACCG
CCTAGGATCC
GGGATTGGCGGCGGTGGTACCTAATGGGACAATAGGGATCCTAGG
Premature termination of SpCas9H-mediated unwinding to the PAM-distal position of more than 17 nt due to the distance of 1st nick
No activation of SpCas9H and no induction of 2nd nick

Figure S7. Possible models explaining the inability of paired *Sp*Cas9n to induce DSBs with 5'-overhangs or 3'-overhangs in the presence of *Trex2* overexpression or not. (a) Upon first nick by *Sp*Cas9^H with gWR3 as an example, first nick is located within or near the second PAM for the second *Sp*Cas9^H-sgRNA. When the second *Sp*Cas9^H-sgRNA unwinds its target and promote base pairing between the target strand and the spacer, the *Sp*Cas9^H-sgRNA-target ternary complex could not be stabilized due to the nick near or within the PAM, thus leading to inefficient induction of second nick. (b) Upon first nick by *Sp*Cas9^D with gEC6 as an example, first nick is induced within the target strand that serves as the nontarget strand for the second *Sp*Cas9^D-sgRNA. When the second *Sp*Cas9^D-sgRNA complex unwind its target with immediate base pairing between the target strand and the spacer of the sgRNA, the *Sp*Cas9^D-sgRNA-target ternary complex might not be fully assembled or activated due to premature termination of the target unwinding upon encountering the nick that is less than 18 nt away on the nontarget strand. Thus, no second nick could be efficiently induced. (c, d) Upon first nick by *Sp*Cas9^H with gWR3 (c) or with gEC6 (d) as an example, first nick is induced within 20 nt towards the PAM for the second sgRNA. When *Sp*Cas9H with the second sgRNA unwinds its target, the *Sp*Cas9^H-sgRNA-target ternary complex might not be fully assembled due to premature termination of the target unwinding upon encountering first nick located on the target strand within 17 nt towards the PAM. Second nick could not be efficiently induced. (e, f) Upon first nick by *Sp*Cas9^H with gEC6 (e) or with gWR3 (f) as an example, the PAM-distal 17-nt 3'-nontarget ssDNA strand released from *Sp*Cas9^H-sgRNA might be attacked by TREX2 overexpressed. At least 5 nt of the PAM-distal 17-nt 3'-nontarget strand might be degraded so that only 17 nt or less are left for unwinding by *Sp*Cas9^H and pairing with the second sgRNA. Consequently, second nick could not be efficiently induced to generate DSBs with 3'-overhangs.

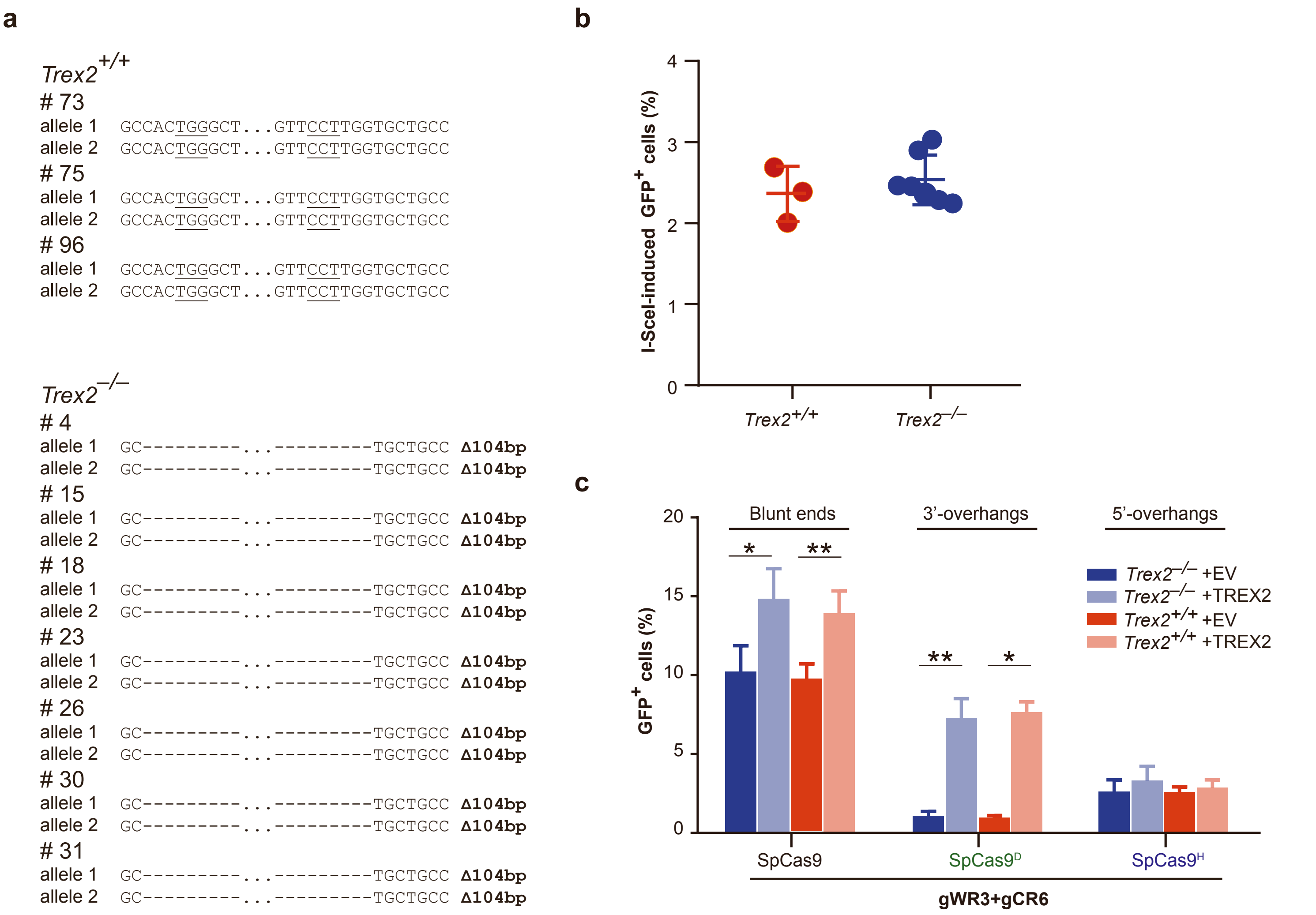

Figure S8. Deletion of endogenous *Trex2* had little effect on m-NHEJ induced by paired *Sp*Cas9n. (**a**) Generation of *Trex2*$^{-/-}$ mouse ES cells containing the m-NHEJ reporter. Reporter mouse ES cells were transfected with expression plasmids for paired *Sp*Cas9-sgRNAs targeting *Trex2* exon 2 and individual clones were selected in about 2 weeks. 7 *Trex2*$^{-/-}$ clones with precise 104-bp deletion in two *Trex2* alleles as well as 3 unedited clones were verified by Sanger sequencing. (**b**) Effect of *Trex2* deletion on m-NHEJ of I-SceI-induced DSBs. Each circle indicates an independent clone. (**c**) Effect of *Trex2* overexpression on m-NHEJ of DSBs induced by *Sp*Cas9-gWR3/gCR6, *Sp*Cas9^{D}-gWR3/gCR6 or *Sp*Cas9^{H}-gWR3/gCR6 in *Trex2*$^{+/+}$ and *Trex2*$^{-/-}$ mouse ES cells. *Trex2*$^{+/+}$ and *Trex2*$^{-/-}$ reporter mouse ES cells were transfected with expression plasmid for *Sp*Cas9-gWR3/gCR6, *Sp*Cas9^{D}-gWR3/gCR6 or *Sp*Cas9^{H}-gWR3/gCR6, together with the EV control or the expression plasmid for *Trex2*. GFP^{+} cells induced were determined 3 days post transfection by FACS, and the frequency of GFP^{+} cells was corrected by transfection efficiency. Columns indicate the mean ± S.E.M from three independent experiments, each in triplicates, and statistics was performed by two-tailed Student’s t-test. *, $P<0.05$; **, $P<0.01$.

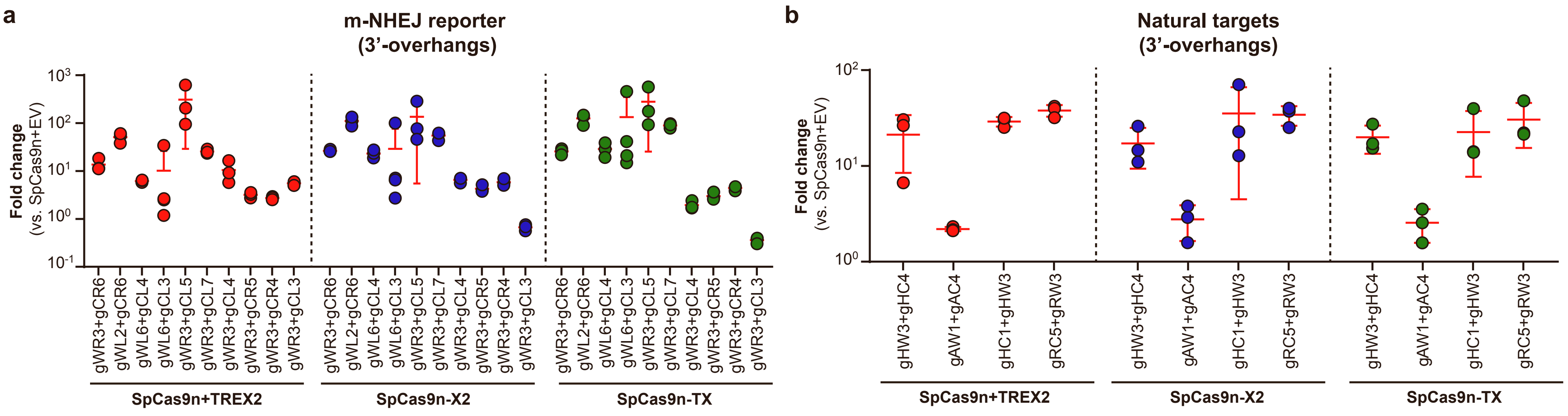

Figure S9. Fold change in stimulation of paired *Sp*Cas9n-induced m-NHEJ by *TREX2* overexpressed and TREX2 fused to *Sp*Cas9n. Paired *Sp*Cas9n-sgRNAs induced DSBs with 3'-overhanging ends at the m-NHEJ reporter in mouse ES cells (**a**) or natural genomic sites in 293T cells and mouse ES cells (**b**) and are indicated on X-axis. The fold change is calculated as the ratio of m-NHEJ induced by paired *Sp*Cas9n-sgRNAs with free *TREX2* overexpressed, paired *Sp*Cas9n-X2-sgRNAs or *Sp*Cas9n-TX-sgRNAs to that by *Sp*Cas9n-sgRNAs with the EV control, respectively. Each circle indicates one independent experiment with triplicates, and the mean of these independent experiments is also shown. Error bars indicate S.E.M.

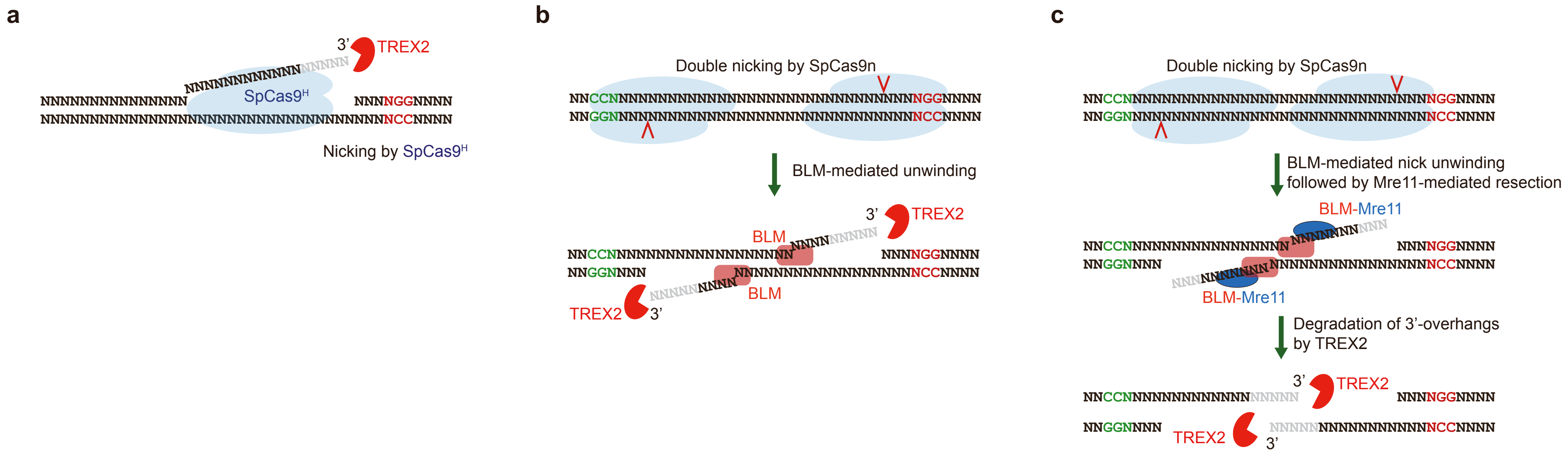

Figure S10. Possible mechanisms underlying generation of 3' ssDNA by paired *Sp*Cas9n and subsequent *TREX2*-mediated degradation of 3' ssDNA. (**a**) The 17-nt 3'-nontarget strand is released from the *Sp*Cas9^{H}–sgRNA-DNA ternary complex and degraded by *TREX2* overexpressed or TREX2 fused to *Sp*Cas9^{H}. (**b**) *TREX2* overexpressed or TREX2 fused to *Sp*Cas9n degrades 3' ssDNA which is generated from unwinding of paired nicks by BLM helicase. (**c**) 3'-overhanging ends are generated from unwinding of paired nicks by BLM in coordination with Mre11-mediated resection. *TREX2* overexpressed or TREX2 fused to SpCas9n can exert its nuclease activity on 3'-overhangs in these 3'-overhanging ends.
